# Specific features of immune ageing are detected in the earliest stages in rheumatoid arthritis development

**DOI:** 10.1016/j.ebiom.2025.105900

**Published:** 2025-09-03

**Authors:** Karim Raza, Archana Sharma-Oates, Leonid Padyukov, Annette H.M. van der Helm-van Mil, Arthur G. Pratt, Simon W. Jones, A. Filer, Janet M. Lord, Niharika A. Duggal

**Affiliations:** a Department of Inflammation and Ageing, School of Infection, Inflammation and Immunology, University of Birmingham, Birmingham, UK; b Department of Rheumatology, Sandwell and West Birmingham NHS Trust, Birmingham, UK; c School of Biosciences, University of Birmingham, Birmingham, UK; d Division of Rheumatology, Department of Medicine Solna, Karolinska Institute, Stockholm, Sweden; e Leiden University Medical Center, Leiden, Netherlands; f Institute of Translational and Clinical Research, Newcastle University, Newcastle-upon-Tyne, UK; g MRC-Versus Arthritis Centre for Musculoskeletal Ageing Research, University of Birmingham, Birmingham, UK; h NIHR Birmingham Biomedical Research Centre, University Hospital Birmingham and University of Birmingham, Birmingham, UK

**Keywords:** Immunesenescence, Rheumatoid arthritis, Immune ageing, Age-related disease

## Abstract

**Background:**

Rheumatoid arthritis is an age-related disease displaying features of an aged immune system. This study aims to determine premature presence of immune ageing in the early stages of RA development, including in patients with clinically suspected arthralgia and undifferentiated arthritis.

**Methods:**

We recruited 224 participants: 69 healthy controls (mean age 57.12 years, 28% male); 32 with clinically suspected arthralgia (mean age 46.50 years, 11% male); 44 with undifferentiated arthritis (mean age 51.96 years, 21% male); 23 with newly presenting DMARD naive RA and 3 months or less symptom duration (mean age 56.5 years, 30% male) and 56 with DMARD naive RA and greater than 3 months symptom duration (mean age 56.41 years, 41% male). Features of immune ageing were assessed via flow cytometry and a subset of 8 immune cell type frequencies were used to generate an integrated score of immune ageing IMM-AGE and transcriptomic analysis for hallmarks of immune ageing was performed.

**Findings:**

Reduced frequencies of naive CD4 T cells and recent thymic emigrants were seen in patients with arthralgia or undifferentiated arthritis. Other features of immune ageing, such as raised frequency of Th17, Tregs and senescent-like T cells, were only seen once RA was established. Overall, the IMM-AGE score and other hallmarks of ageing (inflammation, autophagic defects) were raised in patients during early stages of the disease. Lastly, we have provided evidence of immune ageing features as a predictor of RA development in arthralgia patients.

**Interpretation:**

We have shown that some features of immune ageing are present in the very early stages of RA and may therefore contribute to disease development. Future research should determine whether geroprotective drugs such as spermidine (autophagy booster), senolytics (clearance of senescent cells) and metformin (attenuates inflammation and boosts autophagy) reduce progression of the disease in patients at risk of RA.

**Funding:**

This study was funded by a grant from 10.13039/501100014034FOREUM and the 10.13039/501100008741European League Against Rheumatism (EULAR).

**Research in context**

**Evidence before this study**

Prior research has established that rheumatoid arthritis (RA) is associated with immune alterations indicative of premature immune ageing, particularly in patients with long-standing disease. However, the extent to which premature immune ageing contributes to RA pathogenesis, particularly in the earliest stages before clinical diagnosis, has remained unclear. Most studies have focused on established RA, leaving a gap in understanding whether immune ageing is a driver of disease onset or a consequence of chronic inflammation.

**Added value of this study**

This study provides insights by investigating immune ageing across different RA disease stages, including drug-naïve patients with arthralgia, undifferentiated arthritis, and early RA. It reveals that features of immune ageing, such as reduced naïve T cells, diminished thymic output, and an elevated IMM-AGE score are already present in the preclinical phase drug-naïve patients, suggesting they may contribute to disease development rather than being secondary to chronic inflammation. However, certain features of immune ageing, such as raised frequency of Th17 and senescent-like T cells were only seen once RA was established.

**Implications of all the available evidence**

The findings suggest that immune ageing may serve as a predictive marker for RA development, offering new opportunities for early intervention. Targeting ageing pathways such as autophagy dysfunction, metabolic imbalances, and senescent cell accumulation may provide therapeutic strategies. Anti-ageing interventions, including metformin, spermidine, and senolytics, could be explored as potential disease-modifying treatments in high-risk individuals with preclinical RA.

## Introduction

Advancing age is a major risk factor for several autoimmune inflammatory conditions including rheumatoid arthritis (RA).^1^ Despite the association of RA with age, we understand little of the role ageing processes play in its pathogenesis. It is well established that the remodelling of the immune system occurs with age,^2^ results in increased susceptibility to infections, reduced vaccination responses and compromised immune tolerance.^3^ Key features of immune ageing, such as thymic atrophy,^4^ lymphocyte telomere shortening^5^ and dysregulated Treg and Th17 responses,^6^ have been reported to occur in patients with RA earlier than their healthy peers, predisposing them towards chronicity of inflammation and autoimmunity. Another hallmark of T cell ageing, the accumulation of senescent-like T cells with the characteristic senescence-associated secretory phenotype (SASP) of pro-inflammatory cytokines and chemokines, contributes to inflammaging, the prolonged elevation of basal inflammation levels in older adults.^7^ In parallel with these T cell alterations, B cell ageing is also evident with notable changes including a reduction in naïve B cells, expansion of age-associated B cells^8^ and loss of regulatory B cells.^9^

However, it is not known whether these features of changes in T cell and B cell compartments contribute to RA pathogenesis or are a consequence of the disease. If it is a primary pathogenic factor, then targeting immune ageing could reduce progression from the early stages of RA, such as clinically suspect arthralgia or undifferentiated arthritis, to established RA. Research from Weyand and Goronzy has shown that immune ageing is accelerated by up to 20 years in patients with RA^10^ and biomarkers of T cell immune ageing, such as telomerase insufficiency, are independent of disease activity or duration and are seen in newly diagnosed treatment-naive patients.^11^ These observations suggest the early involvement of immune ageing in the disease process.

The development of a disease classifiable as RA is often preceded by several clinically apparent ‘pre-RA phases’ accompanied by subtle immunological alterations that precede the onset of symptoms. As the diseases progresses patients may experience arthralgia (joint pain) or a state of undifferentiated arthritis (displaying joint pain, swelling or stiffness) prior to clinical manifestation that culminate in the establishment of RA.^12^ In this study, we address whether immune ageing occurs early in disease pathogenesis by assessing immune phenotypes in patients across different stages in the pathway to RA establishment, enabling us to develop a deeper understanding of RA pathogenesis whilst highlighting critical windows for early intervention to alter the disease trajectory.

## Methods

### Patients

Patients were recruited from University Hospital Birmingham and City and Sandwell Hospitals Birmingham to the BEACON cohort of patients with early arthritis. At entry to the cohort, all patients were disease-modifying antirheumatic drug (DMARD)-naïve and had either (i) clinically apparent synovitis of ≥1 joint or (ii) clinical symptoms which in the opinion of the treating Rheumatologist put them at risk of the development of RA (such as morning stiffness, functional impairments indicative of evolving arthritis and serological autoantibody marker levels in the absence of clinically apparent synovitis). Patients were re-assessed after 18 months of follow-up and assigned to an outcome category which included: persistent RA (fulfilling either1987 American College of Rheumatology (ACR) criteria or 2010 ACR/EULAR criteria),^13^ persistent non-RA arthritis and resolving arthritis. Participant demographics such as age, sex, ethnicity were collected and was self-reported by study participants. Clinical measures, including, symptom duration, and Disease Activity Score 28 (DAS28) were also recorded. Frequency matching was performed to balance the age-distribution between patient groups and healthy controls using nearest-neighbour matching with a caliper width of 0.25 standard deviations of the age distribution. Healthy controls were students and staff at the University of Birmingham and older adults recruited from the community who were not suffering any immune-mediated disease or an any immunomodulatory treatments.

### Ethics

The study was approved by the Black Country Research Ethics Committee (REC reference number 12/WM/0258) for the recruitment of clinical patients and University Research Ethics Committee (Ref: ERN_12-1184) for healthy participants. Written informed consent was obtained from all participants prior to inclusion in the study. All procedures involving human participants were conducted in accordance with approvals by the ethics committee.

### Blood cell isolation

Blood samples (12 ml) were collected by venepuncture into BD vacutainers containing lithium heparin and complete blood differential counts were made using a Sysmex haematology analyser. Peripheral blood mononuclear cells (PBMCs) were isolated by density centrifugation using Ficoll–Paque™ PLUS (GE Healthcare, UK; Cat No: GE17-1440-02) and frozen in freezing medium (10% DMSO (Sigma Aldrich, UK; Cat No: D8418) in heat-inactivated foetal calf serum (FCS; Biosera) and stored at −80 °C until further analysis.^14^

### Immune cell phenotyping

**Fig. 1:**
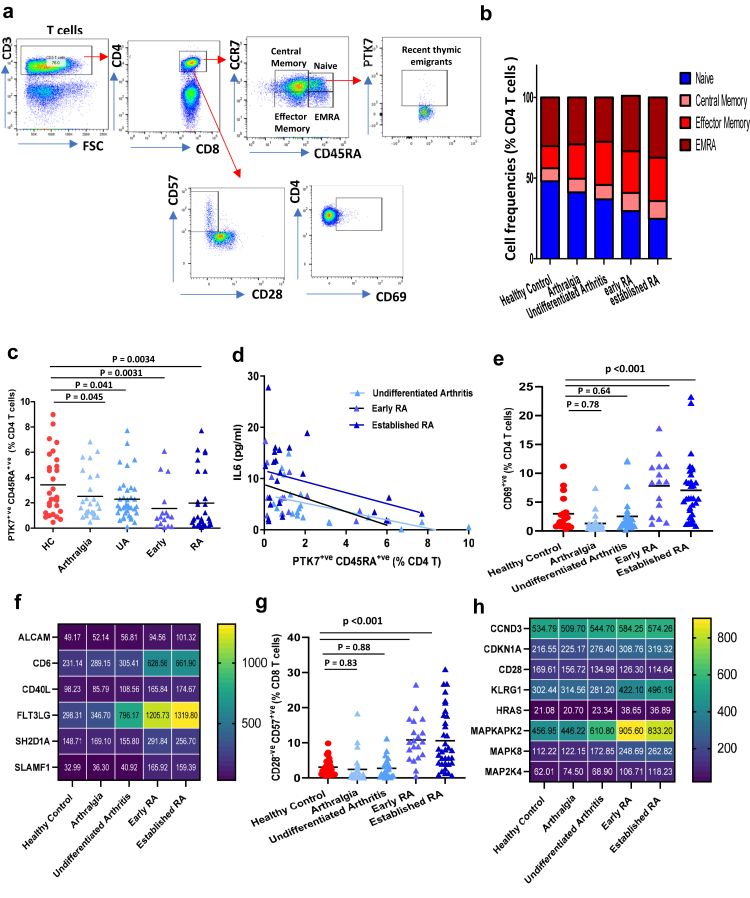
**CD4 T cell subset distribution across disease phases leading up to the development of Rheumatoid Arthritis**. (a) Gating strategy used to analyse markers subsets within CD4^+ve^ and CD8^+ve^ T cells; naïve (CCR7^+ve^CD45RA^+ve^); central memory (CCR7^+ve^CD45RA^−ve^), effector memory (CCR7^−ve^CD45RA^−ve^) and terminal differentiated effector memory re-expressing RA, EMRA (CCR7^−ve^CD45RA^+ve^) T cells. Frequency of (b) Comparison of the proportion of four CD4 T cell subsets across our 5 cohorts; (c) PTK7^+ve^ naïve CD4 T cells (Recent thymic emigrants) in PBMCs isolated from healthy age and sex-matched controls (n = 69), patients with arthralgia (n = 32), undifferentiated arthritis (n = 44), early RA whose disease was confirmed in the last 3 months (n = 23) and established RA whose disease was confirmed at longer than 3 months duration (n = 56). Data represent individual values, mean (centre bar). Statistical analysis was done using a One-way ANOVA to demonstrate significant group differences. (d) Linear regression plot showing correlations (Pearson) between the circulating IL6 levels and peripheral frequency of Recent thymic emigrants in patients with undifferentiated arthritis (n = 20), early RA (n = 18) and established RA (n = 23). The overlaid regression lines were derived using ordinary least squares. Activated CD69^+ve^ CD4 T cells (e) and CD28^−ve^CD57^+ve^ senescent CD4 T cells (g) in PBMCs isolated from healthy age and sex-matched controls (n = 69), patients with arthralgia (n = 32), undifferentiated arthritis (n = 44), early RA (n = 23) and established RA (n = 56). Heat map showing mean expression levels of (f) activation (h) cellular senescence markers in PBMCs isolated from healthy controls (n = 6), patients with arthralgia (n = 6), undifferentiated arthritis (n = 6), early RA (n = 6) and established RA (n = 6). The mean expression value for each group has been indicated inside each box.

Frozen PBMCs were stained for 30 min at 4 °C with combinations of the following antibodies: anti-human CD3 PE cy7 (clone: UCHT1; Thermo Fischer; Cat No:25-0038-42); anti-human CD4 Brilliant violet (clone: RPA-T4; Thermo Fischer; Cat No: 404-0049-42); anti-human CD8 PE (clone:UCHT4; Immunotools; Cat No: 21620084); anti-human CCR7 FITC (clone:150,503; R and D Systems; Cat No: FAB197F-100); anti-human CD45RA APC (clone: HI100; Biolegend; Cat No:304112); anti-human PTK7 PE (clone:188B; Miltenyi Biotech:Cat No: 130-133-755); anti-human CD69 FITC (clone:FN50; Thermo Fischer, UK; Cat No:310903), anti-human CD154 APC (clone:SA047C3; Thermo Fischer Cat No: 310810); anti-human CD28 APC (clone:CD28.2; BD Biosciences; Cat No:559770) and anti-human CD57 FITC (clone:HCD5; Thermo Fischer; Cat No: 359604). A viability dye Near-IR 635 nm (Thermo Fischer; Cat No: L10119) was used to gate out dead cells. CD3^+^ cells were defined as T cells, 10,000 T cells were gated and were divided into CD4^+^ and CD8^+^, which were further divided into four subsets based on CD45RA and CCR7 expression [gating strategy, Fig. 1a]. The CD69^+^ and CD154^+^ were denoted as activated T cells. CD3^+ve^CD28^−ve^CD57^+ve^ cells were denoted as senescent T cells.^14^ Absolute numbers of immune cell subsets were calculated by multiplying the frequency of each subset by absolute lymphocyte count obtained from complete blood count analysis.

For regulatory T cells and Th17 cells, PBMCs were stained with anti-human CD3 PE cy7, anti-human CD4 Violet for 30 min at 4 °C, fixed with Foxp3 Fix Perm solution (Thermo Fischer; Cat No:00-5523-00) for 30 min at room temperature and staining with anti-human Foxp3 PE (clone: PCH101; Thermo Fischer; Cat No: 12-4776-42) and anti-human RORγ APC (clone: AFKJS-9; Thermo Fischer; Cat No: 17-6988-82) in diluted permeabilization buffer (Thermo Fischer) for 30 min at 4 °C. 5000 CD4 T cells were gated and were divided into Foxp3^+^ and RORγ^+^ cells [gating strategy, Fig. 3a].

**Fig. 2:**
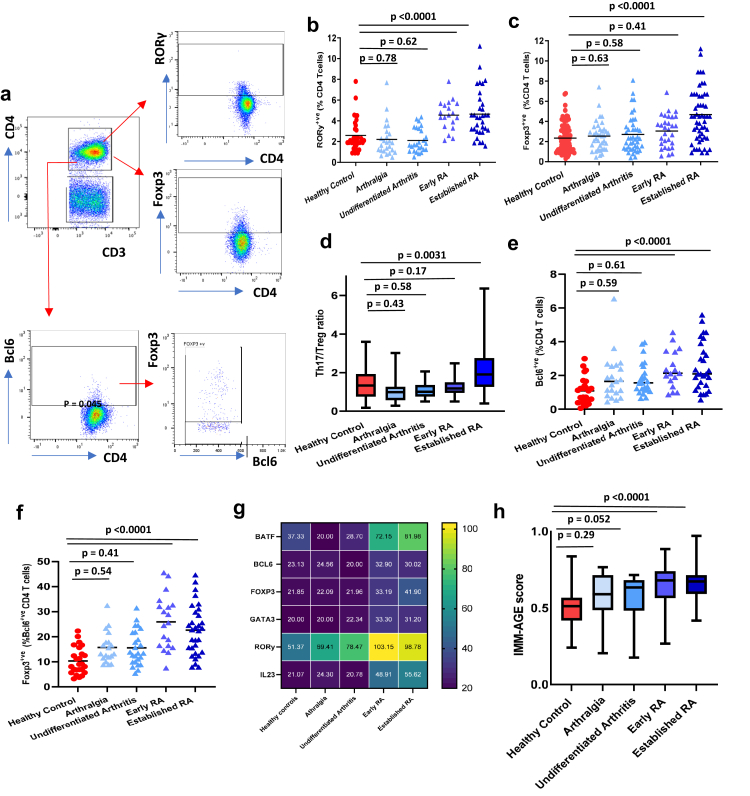
**CD4 helper subset distribution and Immunological ageing (IMM-Age) score across disease phases leading up to the development of Rheumatoid Arthritis**. (a) Gating strategy used to analyse markers subsets within CD4^+ve^ T cells; Th17 (RORγ^+ve^); Regulatory (Foxp3^+ve^), Follicular Helper (Bcl6^+ve^) and Follicular regulatory (Foxp3^+ve^Bcl6^+ve^) T cells. Frequency of (b) Th17 cells (c) Regulatory T cells (d) Th17/Treg ratio (e) Follicular helper (f) Follicular helper regulatory T cells in PBMCs isolated from healthy age and sex-matched controls (n = 69), patients with arthralgia (n = 32), undifferentiated arthritis (n = 44), early RA whose disease was confirmed in the last 3 months (n = 23) and established RA whose disease was confirmed at longer than 3 months duration (n = 56). Data represent individual values, mean (centre bar). Statistical analysis was done using a One-way ANOVA to demonstrate significant group differences. (g) Heat map showing mean expression levels of transcription factors linked with CD4 helper T cell differentiation in PBMCs isolated from healthy controls (n = 6), patients with arthralgia (n = 6), undifferentiated arthritis (n = 6), early RA (n = 6) and established RA n = 6). (h) IMM-AGE scores calculated in healthy age and sex-matched controls (n = 69), patients with arthralgia (n = 32), undifferentiated arthritis (n = 44), early (n = 23) and established RA (n = 56). Statistical analysis was done using a One-way ANOVA to demonstrate significant group differences. In box whisker plots the bars represent mean of the data set and error bars indicate the data variability.

Commercially available antibodies used for flow cytometry were validated using positive and negative cell populations, titration curves, and isotype controls to confirm specificity and optimal staining conditions.

### Immune ageing estimation

A subset of 8 immune cell types (total T cells, naive CD4 T cells, effector memory CD4 and CD8 T cells, EMRA CD8 T cells, CD28^−^ CD8 T cells, CD57^+^ CD8 T cells and regulatory T cells) were used to generate a score for the degree of immune ageing, based on the modified IMM-AGE algorithm,^15^ as previously reported.^16^

### Serum cytokine analysis–Luminex

Blood collected in anti-coagulant free BD vacutainers was left upright for 30 min prior to centrifugation at 1620×*g* for 10 min at room temperature, after which the serum was removed and stored at −80 °C prior to analysis. The concentration of serum cytokines (IL1β, IL4, IL6, IL7, IL10, IL17, TNFα, IFNγ, CRP and CXCL9) was determined using a Human Premixed Multi-Analyte Magnetic Luminex Assay (R&D Systems).

### RNA isolation and Nanostring nCounter gene expression analysis

Total RNA was isolated from 2 × 10^6^ PBMCs from healthy controls and patients using the RNeasy Mini isolation kit (Qiagen, Germany). RNA concentrations and quality were measured using the Agilent 2100 BioAnalyzer. Gene expression analysis was performed using the Pan-Cancer Immune Profiling Panel from NanoString technologies (NanoString, USA). For each sample, 80 ng of total RNA, with a maximum of 7 μL (>28.6 ng/μL), was used. Hybridisation was performed at 65 °C for 17 h using a SimpliAmp Thermal Cycler (Applied Biosystems, UK). The nCounter Flex system (NanoString, USA) was used for sample preparation. Raw gene counts were normalised using the most stable housekeeping genes from the panel. Differential expression of genes between PBMC from the two cohorts was tested with Mann–Whitney U tests and Benjamin-Hochberg procedures were used to correct for multiple testing. The BH false discovery method was used and a p-value cut-off of <0.05 was set as significant. Fold changes between groups were calculated as the ratio of mean expression levels. We calculated 95% confidence intervals (CIs) for fold changes using the log-transformed values. First, we computed the log fold change (LFC) and its standard error (SE). The 95% CI for the LFC was calculated as: [LFC] ±1.96 × [SE]. We then exponentiated the lower and upper bounds to return to the original scale to calculate the 95% CI for the fold change.

### Statistical analysis

All statistical analyses were performed using GraphPad Prism software version 9. Data distribution was examined using Kolmogorov–Smirnov normality test and using graphical methods by generating normality plots allowing the visual inspection of deviations from normality. Next, we assessed homogeneity of variance using the Leven’s test. For normally distributed data and when homogeneity of variance was confirmed, a student t-test, or a one-way ANOVA with Bonferroni multiple comparison post hoc test was performed where appropriate and p ≤ 0.05 was used to define statistical significance. If normality was not met or homogeneity of variance was violated, we applied the Welch’s ANOVA. Multiple linear regression was performed to test for associations between immune parameters and other variables that were selected based on their biological relevance to the study hypothesis and the model was constructed using the Enter method. Linearity was evaluated through scatter plots of continuous predictors and outcome, and normality of residuals was assessed using the Kolmogorov–Smirnov test. Regarding data completeness for participant demographics and clinical data, we have complete data for age, sex, DAS28 scores, and ACPA status for all participants in our study and a complete data set for immunological age IMM-AGE score as well. Ethnicity data were available for the majority of participants, with missing information for less than 4% of the participants.

### Role of funders

The funders had no role in study design, data collection, data analyses, interpretation, or writing of the manuscript.

## Results

### Participant demographics and clinical characteristics

A total of 224 participants were recruited to the study, 69 age and sex matched healthy controls and patients with either arthralgia (n = 32), undifferentiated arthritis (n = 44), confirmed RA within the first 3 months of symptom duration (Early RA; n = 23) and more established disease of greater than 3 months of symptom duration (Established RA; n = 56). The details of the cohort are shown in Table 1.

**Table 1:** Participant demographics and clinical parameters.

	Healthy controls (n = 69)	Arthralgia (n = 32)	UA (n = 44)	Early RA (n = 23)	Established RA (n = 56)
Age: years (mean ± S.D)	57.12 ± 14.19	46.50 ± 14.17	51.96 ± 15.67	56.52 ± 14.55	56.41 ± 16.50
Males [n (%)]	28 (40.6%)	11 (34.3%)	21 (47.8%)	7 (30.4%)	23 (41.1%)
Ethnicity (%Caucasian)	47 (68.1%)	20 (62.5%)	26 (59.1%)	17 (73.9%)	36 (47.5%)
Symptom duration, weeks median (IQR)	n/a	50 (24–96)	48 (20–104)	8 (4–12)	64 (36–88)
RF positive [n (%)]	n/a	10 (31.3%)	6 (13.6%)	14 (60.9%)	25 (44.6%)
ACPA positive [n (%)]	n/a	14 (43.7%)	4 (9.1%)	15 (65.2%)	24 (42.8%)
Smoking status—current/ever [n (%)]	0	18 (56.2%)	20 (45.5%)	12 (52.2%)	24 (42.8%)
Alcohol (units/weeks)	3 (1–5)	5 (2–10)	6 (2–12)	5 (1–11)	6 (5–9)
Body mass index (kg/m^2^)	25.57 ± 4.89	29.57 ± 6.32	27.72 ± 5.49	28.78 ± 4.67	27.69 ± 5.39
DAS 28_ESR scores	n/a	3.30 ± 1.51	3.46 ± 1.58	4.89 ± 1.12	5.32 ± 1.56
DAS 28_CRP scores	n/a	3.24 ± 1.24	3.51 ± 2.39	4.67 ± 1.05	4.98 ± 1.42

Data are mean ± standard deviation is reported for variables with reasonably symmetrical distribution, and median (IQR) for variables with skewed distribution including those taking positive values with mean/SD < 2.

ACPA, Anti-Citrullinated Protein Antibodies; DAS, Disease Activity Score.

### T cell frequency and phenotype

Within the CD4 T cell pool, the frequency and absolute numbers of circulating naïve T cells were lower in arthralgia patients (p = 0.031), undifferentiated arthritis (p = 0.0023) and were maintained in those with diagnosed RA of more or less than 3 months symptom duration (both “p < 0.0001”) [Fig. 1b; Supplementary Table S1]. Peripheral recent thymic emigrants (RTE), cells that have recently left the thymus, were identified using PTK7 and CD45RA.^17^ We also observed a significantly lower frequency of RTEs in participants with arthralgia (p = 0.045), undifferentiated arthritis (p = 0.041), and both early (p = 0.0031) and established (p = 0.003) RA in comparison with healthy controls [Fig. 1a and c]. In this study, we observed a significant association between pro-inflammatory cytokine IL-6 levels and circulating RTEs in undifferentiated arthritis (p = 0.019, adjusted R^2^ = 0.30), early RA (p = 0.016, adjusted R^2^ = 0.30) and established RA (p = 0.021, adjusted R^2^ = 0.29) patients [Fig. 1d], but not in arthralgia patients (p = 0.63, adjusted R^2^ = 0.14; data not shown).

Unsurprisingly the reduction in naïve T cells was balanced by an increase in increased frequency and absolute number of central memory (p = 0.043 and p = 0.0021 respectively) and terminally differentiated EMRA memory CD4 T cells (p = 0.041, p = 0.0033 respectively) in early and established RA compared to healthy controls [Fig. 1b; Supplementary Table S1]. Lastly, an accumulation of effector memory CD4 T cells was observed in all four groups of patients, “p < 0.001” compared to healthy controls [Fig. 1b; Supplementary Table S1]. Within the CD8 T cell pool similar changes were observed [Supplementary Fig. S1a and Table S2].

### Activated and senescent-like T cells

We found enrichment of CD69, an early activation marker [Fig. 1e and CD154, a marker of antigen-specific stimulation expressing CD4 T cells [data not shown] and CD8 T cells [Supplementary Fig. S1b and c] in both patients with early and established (“p < 0.0001”) in comparison with healthy controls, but not in arthralgia (p = 0.78) and undifferentiated arthritis patients (p = 0.64). Similarly, higher protein expression levels of CD69 and CD154 were observed in patients with established RA [Fig. 1e]. The binding of CD6-ALCAM complex helps stabilise the immunological synapse between the T cell and the antigen-presenting cell to promote T-cell activation, maturation, and trafficking into tissues.^18^ We detected a higher gene expression of CD6 and ALCAM and fms-related tyrosine kinase 3 FLT3 ligand, and SH2D1A encoded signalling lymphocyte activation molecule (SLAMF1) in PBMCs of patients with early and established RA [Fig. 1f]. Next, an increase in the frequency and absolute numbers of these senescent-like CD28^−ve^ CD57^+ve^ CD4 T cells was only observed in the patients with early and established RA [“p < 0.001”; Fig. 1g and Supplementary Table S1, but not in arthralgia (p = 0.83) and undifferentiated arthritis patients (p = 0.88). Importantly, we saw increased expression of cellular senescence markers (gain of KLRG1 and loss of CD28) and accelerators of cell cycle arrest (CDKN1A, CCND3) and senescence (HRAS)^19^ in patients with early and established RA [Fig. 1h]. A key trait of these senescent-like T cells is the secretion of pro-inflammatory (e.g., TNFα, IL-6) and tissue remodelling factors, termed senescence-associated secretory phenotype (SASP), implemented by Mitogen-activated protein kinases (MAPKs) which may contribute towards the inflammaging observed in patients with RA [Table 2].

**Table 2:** Systemic cytokine levels.

	Healthy controls (n = 43)	Arthralgia (n = 22)	UA (n = 22)	Early RA (n = 15)	Established RA (n =29)	p value
IL1ß (pg/ml)	0.74 ± 0.66	7.87 ± 6.32	6.07 ± 4.72	9.72 ± 5.46	14.69 ± 11.06	**<0.001**
IL4 (pg/ml)	2.34 ± 0.48	1.98 ± 0.93	3.4 ± 1.08	1.96 ± 0.46	2.11 ± 1.23	0.47
IL6 (pg/ml)	1.88 ± 1.65	5.28 ± 5.93	10.54 ± 6.48	14.96 ± 0.28	22.11 ± 19.23	**0.03**
IL7 (pg/ml)	7.66 ± 3.69	9.09 ± 5.28	8.37 ± 5.90	8.39 ± 5.27	6.42 ± 5.44	0.54
IL10 (pg/ml)	3.51 ± 1.92	4.39 ± 2.84	5.25 ± 2.14	9.57 ± 6.12	8.45 ± 5.50	**0.04**
IL17 (pg/ml)	1.31 ± 0.63	3.52 ± 3.44	5.24 ± 3.39	10.77 ± 8.82	16.51 ± 11.23	**<0.001**
TNFα (pg/ml)	3.54 ± 1.24	8.91 ± 7.62	8.6 ± 7.09	16.42 ± 17.03	34.24 ± 22.38	**<0.001**
CRP (μg/ml)	1.20 ± 0.55	9.69 ± 4.56	10.59 ± 11.99	21.17 ± 19.22	23.57 ± 15.75	**<0.001**
IFNγ (pg/ml)	145.7 ± 48.4	186.6 ± 86.07	171.05 ± 93.3	231.2 ± 53.22	189.53 ± 75.54	0.67
CXCL9 (pg/ml)	2704 ± 1503	3252 ± 2598	3992 ± 1617	4875 ± 2034	4556 ± 3080	**0.04**

The significant data is bold.

### Helper T cell subset distribution

Th17 cells regulated by transcription factor retinoic acid receptor-related orphan receptor-γt (RORγt), are capable of inducing autoimmunity.^20^ We found a raised frequency of pro-inflammatory Th17 cells in patients with early and established RA (“p < 0.0001”), but not in those with arthralgia (p = 0.78) or undifferentiated arthritis (p = 0.62), in comparison with healthy controls [Fig. 2b]. We also detected a higher gene expression of RORγt and IL23 and BATF known inducers of Th17 differentiation^21^ in patients with early and established RA [Fig. 2g]. Regulatory T cells characterised by the expression of Foxp3, play an immunoprotective role in RA through multiple mechanisms, including cell-to-cell contact, and the secretion of the regulatory cytokine IL-10.^22^ We detected an increase in the peripheral frequency of T_regs_ in patients with established RA with symptom duration of >3 months (p < 0.0001) [Fig. 2c] and higher expression levels [Fig. 2g], but not in the patients with arthralgia (p = 0.63) or undifferentiated arthritis (p = 0.58), or early RA (p = 0.41) in comparison with healthy controls [Fig. 2c]. Overall, the Th17/T_reg_ balance was disturbed upon the establishment of RA and was significantly higher when compared to healthy controls (p = 0.0031), but not in patients with arthralgia (p = 0.43), undifferentiated arthritis (p = 0.58) or early RA (p = 0.17) [Fig. 2d].

**Fig. 3:**
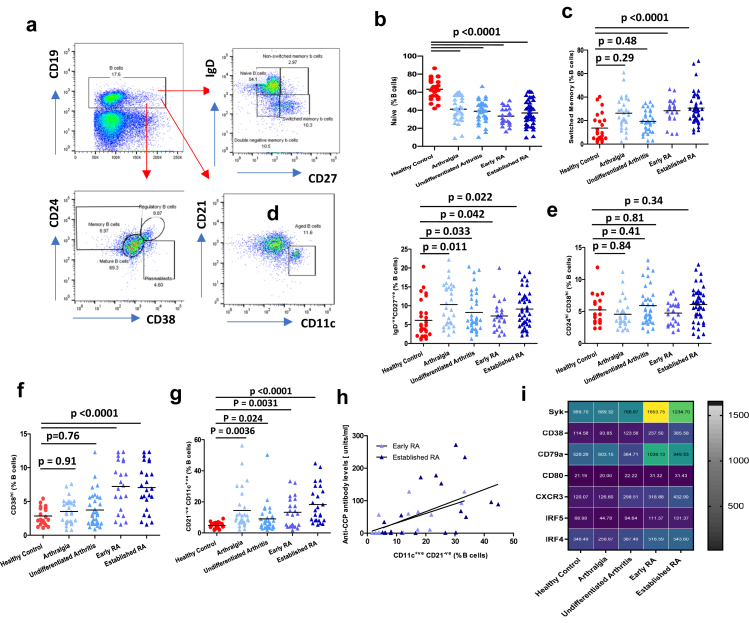
**B cell subset distribution across disease phases leading up to the development of Rheumatoid Arthritis**. (a) Gating strategy used to analyse markers subsets within B cells; naïve (CD27^−ve^IgD^+ve^); unswitched memory (CD27^+ve^IgD^−ve^), switched memory (CD27^+ve^ IgD^+ve^) and double negative (CD27^−ve^ IgD^−ve^) B cells. Regulatory CD38^hi^ CD24^hi^ B cells, CD38^hi^CD24^−ve^ Plasmablasts and CD21^−ve^CD11c^+ve^ age associated B cells. Frequency of (b) Naive (c) Switched Memory (d) Unswitched memory (e) Regulatory (f) Plasmablasts (g) Age-associated B cells in PBMCs isolated from healthy age and sex-matched controls (n = 62), patients with arthralgia (n = 29), undifferentiated arthritis (n = 40), early RA whose disease was confirmed in the last 3 months (n = 20) and established RA whose disease was confirmed at longer than 3 months duration (n = 51). Data represent individual values, mean (centre bar). Statistical analysis was done using a One-way ANOVA to demonstrate significant group differences. (h) Linear regression plot showing correlations (Pearson) between the circulating anti-ACPA levels and peripheral frequency of Age Associated B cells in patients with early RA (n = 18) and established RA (n = 23). The overlaid regression lines were derived using ordinary least squares (i) Heat map showing mean expression levels of B cell markers in PBMCs isolated from healthy controls (n = 6), patients with arthralgia (n = 6), undifferentiated arthritis (n = 6), early (n = 6) and established RA (n = 6).

Follicular helper T (Tfh) cells facilitate the generation of long-lived antibody-secreting plasma cells and play a critical role in RA pathogenesis.^23^ We detected an increase in the peripheral frequency of Tfh cells in patients with early and established RA (“p < 0.0001”), in comparison with healthy controls, but not in patients with arthralgia (p = 0.59) and undifferentiated arthritis (p = 0.61) [Fig. 2e]. A similar expansion of follicular regulatory (Tfr) cells a sub-population of Foxp3 expressing Tfh cells that can migrate to the germinal centre and inhibit Tfh-mediated B-cell antibody production^24^ was observed in patients with early and established RA (“p < 0.0001”), but not in patients with arthralgia (p = 0.54) and undifferentiated arthritis (p = 0.41) [Fig. 3f].

### IMM-AGE score for immunological age

We calculated the IMM-AGE score, an indication of the overall degree of ageing of the immune system,^15^^,^^16^ for each of the patient groups. The data show a significantly higher immunological age in patients with undifferentiated arthritis (p = 0.052), which is further increased in patients with early and established RA (“p < 0.001” for both) compared to healthy age-matched controls. IMM-AGE was not raised in patients with arthralgia (p = 0.29) [Fig. 3h]. To try and understand to the extent to which higher IMM-AGE scores reflected pre-existing immune ageing, we carried out multiple linear regressions considering variables that could affect the score namely BMI, ethnicity, age, sex, disease duration and severity [Supplementary Tables S3–S6]. The analysis revealed the only pre-existing variable influencing the IMM-AGE score in the undifferentiated arthritis patients was the sex (male) (β = 0.39, p = 0.0072) [Supplementary Table S4]. In patients with established disease, smoking status was observed to influence the IMM-AGE score β = 0.32, p = 0.047 [Supplementary Table S5].

### B cell subset distribution

**Fig. 4:**
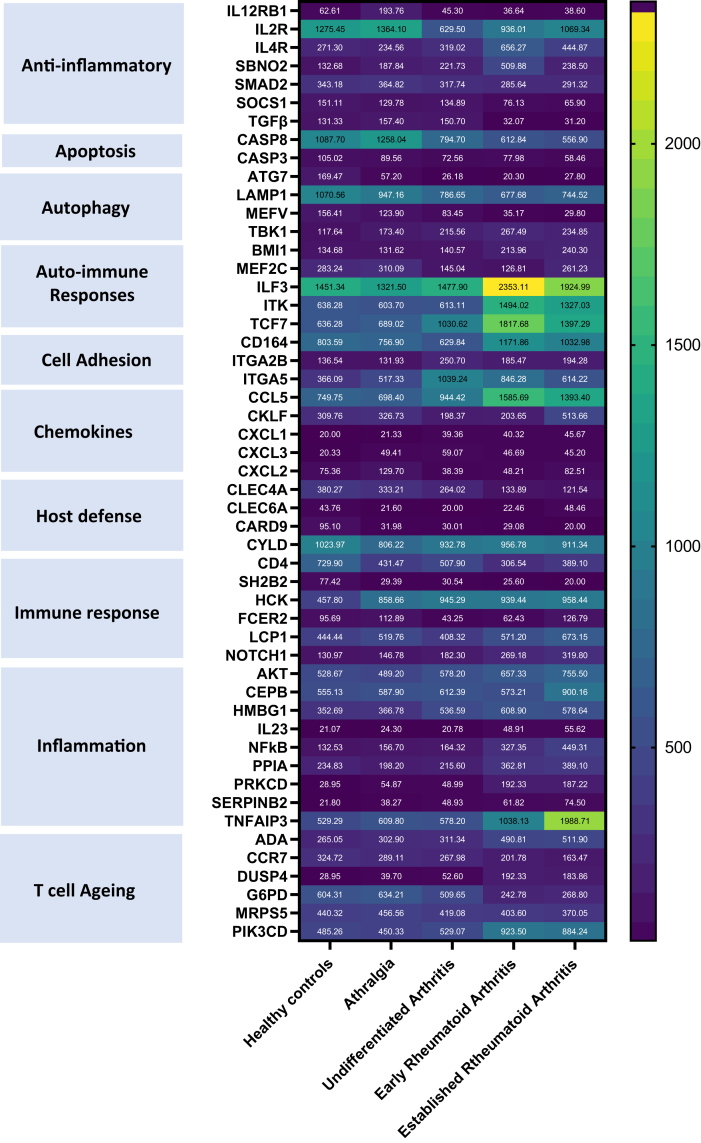
**Transcriptome signature across disease phases in rheumatoid arthritis**. A heatmap showing the relative expression levels of a selection of significantly expressed genes in PBMCs isolated from healthy controls (n = 6), patients with arthralgia (n = 6), undifferentiated arthritis (n = 6), early RA (n = 6) and established RA (n = 6). The gene IDs can be seen on the X axis. The figure legend colour corresponds to the relative expression levels of a given gene within a group.

On assessing total B cell frequencies and absolute numbers, no significant differences were observed between the groups [Supplementary Table S7]. Within the B cell pool, using the IgD/CD27 classification we have identified four subsets^25^ [Gating strategy Fig. 4a]. The frequency and absolute numbers of circulating naïve B cells [IgD^+ve^ CD27^−ve^] were lower in patients with arthralgia, undifferentiated arthritis and patients diagnosed with rheumatoid arthritis, p < 0.0001 [Fig. 3b; Supplementary Table S7]. Next, an expansion of switched memory B cells was observed in patients with early and established RA “p < 0.0001”, but not in patients with arthralgia (p = 0.29) and undifferentiated arthritis (p = 0.48) [Fig. 3c; Supplementary Table S7]. Lastly, we observed an expansion of pro-inflammatory aged IgD^−ve^ CD27^−ve^ double negative B cells, that express the highest SASP features amongst memory B cells,^26^ in patients with, undifferentiated arthritis and patients diagnosed with rheumatoid arthritis (p = 0.0031) in comparison with healthy controls [Fig. 3d; Supplementary Table S7].

Regulatory B-cells (CD24^hi^CD38^hi^) play a vital role in controlling autoimmune diseases such as RA, mediated via secreting anti-inflammatory cytokines including IL10 to restrain inflammation and promote the differentiation of immunoregulatory T cells and inhibit the differentiation of Th17 cells.^27^ The analysis of circulating levels of regulatory B cells revealed no statistically significant differences between patients with arthralgia (p = 0.84), undifferentiated arthritis patients (p = 0.41), patients with early (p = 0.81) and established RA (p = 0.34) compared to healthy controls [Fig. 3e; Supplementary Table S7]. However, an expansion of CD38^hi^ CD24^lo^ plasmablasts was observed in patients with early and established RA “p < 0.0001”, but not in patients with arthralgia (p = 0.91) and undifferentiated arthritis (p = 0.76) [Fig. 3f; Supplementary Table S7]. An increased expression of IRF4 that plays a fundamental role in B cell differentiation towards antibody-secreting plasma cells was also seen in patients with early and established RA.^28^ Furthermore, we have observed an upregulation of the B cell lineage marker CD79b, activation marker CD80, and chemokine receptor CXCR3, which plays a crucial role in the homing of B cells to an inflammatory environment, and activation of SYK (spleen tyrosine kinase) pathways [Fig. 3i]; known contributors towards disease pathogenesis.^29^

Autoantibody secreting Age-associated B cells (ABCs) expressing CD11c and displaying a reduced expression of CD21 expanded with autoimmune conditions are known to play a role in promoting disease pathogenesis.^30^ In this study, we report that an expansion of these ABCs is an early feature of disease and can be seen in patients with arthralgia and undifferentiated arthritis, p = 0.0036 and p = 0.02 respectively, which is further expanded in patients with early, p = 0.0031 and established RA “p < 0.0001” [Fig. 3g]. Interestingly, we have observed a positive association between serum anti-CCP antibody levels and peripheral frequency of ABCs in patients with early [adjusted R^2^ = 0.27, p = 0.029] and established [adjusted R^2^ = 0.40, p = 0.012] RA [Fig. 3h].

### Inflammation from arthralgia to RA

In this study, we observed that systemic inflammation preceded RA disease establishment and elevated levels of pro-inflammatory cytokines IL-1beta (p = 0.0042), IL-6 (p = 0.021), TNFα (p = 0.03) and CRP (p = 0.032) were higher in patients with arthralgia compared to healthy controls which was further elevated upon disease establishment. Cytokines, such as IL-17 play a role in RA disease pathogenesis and elevated levels of circulating IL17 were observed in patients with early (p = 0.0012) and established (“p < 0.001”) RA; similar to the expansion of the Th17 subset [Supplementary Table S8]. A new inflammatory ageing clock (iAge) has been recently proposed to be an accurate metric for multimorbidity that can be used for early detection of an age-related clinical phenotype. A major contributor to iAge was chemokine CXCL9 secreted by immune cells to attract them to the site of infection.^31^ In this study we have observed elevated levels of CXCL9 only in patients with early (p = 0.009) and established RA (p = 0.032) [Supplementary Table S8].

Anti-inflammatory cytokines such as interleukin IL-10 and IL-4 are known for their regulatory activity to restore immune homoeostasis and prevention of joint degradation. Elevated systemic levels of circulating IL-10 were observed in both patients with early (p = 0.03) and established (p = 0.052) RA, but no-significant differences were observed in IL-4 levels [Supplementary Table S8].

### Transcriptome signature

Next, to elucidate molecular signalling pathways contributing toward this state of enhanced immune ageing we used the Nanostring nCounter gene expression assay allowing for the detection of 770 genes in PBMCs from six healthy controls, patients with arthralgia, undifferentiated arthritis, early and established arthritis all of caucasian ethnicity non-smokers with a healthy BMI and were age-sex matched [Supplementary Tables S9–S12]. A heatmap was generated by clustering analysis of the differentially expressed genes [Fig. 4]. Firstly, we observed downregulation of anti-inflammatory genes such as SMAD2 and SOCS1 that downregulates inflammatory cytokine secretion by inhibiting JAK/STAT pathway in patients with RA, but not during early disease stages. On the contrary, we have observed an upregulation of inflammation promoting mediators (e.g., NF-kB, AKT) and inflammatory cytokines (e.g., IL23 that enhances expansion of Th17 cells) only in patients with RA [Fig. 4].

**Fig. 5:**
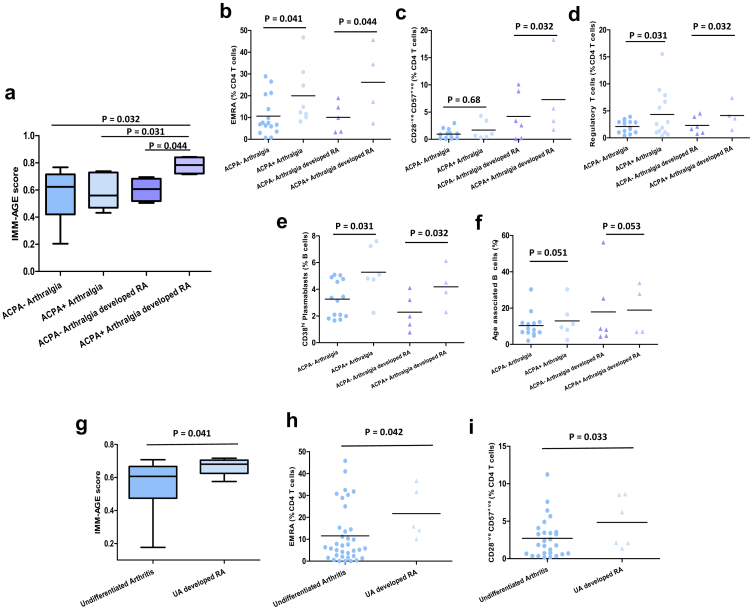
**Accelerated immunological ageing in arthralgia and undifferentiated arthritis patients developing RA**. (a) IMM-AGE scores. Peripheral frequency of (b) EMRA CD4 T cells (c) CD28^−ve^ CD57^+ve^ senescent CD4 T cells (d) Foxp3^+ve^ regulatory CD4 T cells (e) CD38^hi^ plasmablast B cells (f) CD11c^+ve^ CD21^−ve^ age associated B cells in ACPA^−ve^ patients with arthralgia (n = 14), ACPA^+ve^ patients with arthralgia (n = 6), ACPA^−ve^ patients with arthralgia that developed RA (n = 6) and ACPA^+ve^ patients with arthralgia that developed RA (n = 4). Statistical analysis was done using a One-way ANOVA to demonstrate significant group differences. (g) IMM-AGE scores. Peripheral frequency of (h) EMRA CD4 T cells (i) CD28^−ve^ CD57^+ve^ senescent CD4 T cells in patients with undifferentiated arthritis (n = 38), and those patients with undifferentiated arthritis that develop RA (n = 6). Statistical analysis was done using a One-way ANOVA to demonstrate significant group differences.

Furthermore, we found an upregulation of expression of autoimmunity associated genes in patients with RA, such as BM1 that is expressed in antibody secreting cells, interleukin enhancer-binding factor 3 (ILF3), a known autoantigen that is only seen in patients with RA and TCF1 (encoded by *TCF7*) a transcription factor known to contribute to the perpetuation of the autoimmune response. We have observed a reduction of autophagy related genes (e.g., ATG7 that is necessary for LC3 processing and conjugation) [Fig. 5] during the early stages of the disease hinting at a role of autophagy in disease development by contributing towards apoptosis resistance (e.g., caspase 3 expression reduced) and impaired elimination of antigens and possibly contributing towards the accumulation of inflammatory cells.^32^

Lastly, we saw an increased expression of specific metabolic reprogramming markers such as increased Dual-Specific Phosphatase 4 (DUSP4)^33^ and loss of Glucose-6-phosphate dehydrogenase (G6PD) in patients with RA [Fig. 5] driving an aged phenotype in T driving their arthritogenic properties.

### Immune biomarkers to identify arthralgia and undifferentiated arthritis patients at risk of developing RA

In this study we observed a 28% progression from arthralgia to arthritis over a 24-month time follow up period and out of the 9 patients who developed RA; 4 were ACPA^+ve^ and 5 were ACPA^−ve^. On assessment of the potential of IMM-AGE score to identify patients at risk of developing RA in the pre-arthritis phase, a significantly higher IMM-AGE score was observed in ACPA^+ve^ patients with arthralgia (p = 0.032) who developed RA compared to ACPA^−ve^ patients with arthralgia and those who do not develop RA at the baseline stage [Fig. 5a].

Furthermore, seropositive arthralgia patients display an array of altered cytokines and immune profile especially compared with seronegative (arthralgia) patients [Supplementary Table S13]. We have observed a significant increase in frequency of EMRA CD4 T cells and regulatory T cells in ACPA^+ve^ patients with arthralgia irrespective of whether they developed RA or not, p = 0.041 and p = 0.032 respectively compared to ACPA^−ve^ arthralgia patients [Fig. 5b; Fig. 5c]. Perhaps unsurprisingly, autoantibody secreting plasmablasts were expanded in ACPA^+ve^ patients with arthralgia in both non-converters (p = 0.031) and converters (p = 0.032) compared to ACPA^−ve^ arthralgia patients [Fig. 5e]. Lastly, we observed an expansion of age-associated B cells in ACPA^+ve^ (p = 0.051) and ACPA^−ve^ (p = 0.053) patients with arthralgia who developed RA compared to those who did not develop RA [Fig. 5f].

### Immune biomarkers to identify undifferentiated arthritis patients at risk of developing RA

Next, we assessed biomarkers to identify patients diagnosed with undifferentiated arthritis at an increased risk of developing RA [Supplementary Table S14]. In our cohort only four patients developed rheumatoid arthritis. Of the 4 patients with undifferentiated arthritis that were ACPA positive, 2 developed RA. On assessment of the potential of IMM-AGE score to identify patients with undifferentiated arthritis at risk of developing RA, we observed a significantly higher score in these patients that develop RA [p = 0.041; Fig. 5g]; driven by an expansion of terminally differentiated effector memory [p = 0.042; Fig. 5h] and senescent-like CD4 T cells [p = 0.033; Fig. 5i].

### Longitudinal assessment of effect of established therapies in combatting features of accelerated immune ageing in patients with RA

Patients with newly diagnosed RA are started on disease modifying anti-rheumatic drugs (DMARDs) such as methotrexate to reduce joint inflammation, and the risk of progressive joint damage.^34^ Whilst the Disease Activity Scores (DAS_CRP) declined in patients with RA over the first 6 months (p = 0.032), our pilot immunological analysis revealed no changes in immunological ageing features in these patients over the first 6 months after starting DMARDs [Supplementary Table S15].

## Discussion

Our study set out to explore whether premature immune ageing may contribute to RA pathogenesis or be a consequence of the disease itself. Multiple studies have reported immune alterations indicative of immune ageing in patients with established RA displaying heterogeneity regarding disease durations and prescribed therapies.^35^ We have investigated immunological, inflammatory and gene expression signatures across different disease stages in drug-naïve patients, revealing a greater degree of immune ageing in the early stages of disease which increases in patients with established disease. Specifically, features such as reduced numbers of naive T cells and RTEs, as well as a raised IMM-AGE score and an inflammatory milieu (elevated systemic IL6, TNFα and CRP levels) are seen in patients with arthralgia and undifferentiated arthritis that may be contributing to creating a pre-disease microenvironment, but other features such as accumulation of senescent-like T cells are only seen at RA development.

A state of premature diminution of thymic production has been previously observed in patients diagnosed with RA.^36^ In this study, we report a decline in thymic output at early stages prior to disease establishment. Although statistically significant the mean difference in circulating frequencies of recent thymic emigrants between the groups was relatively small; suggesting that it alone may not serve as robust clinical biomarker. The molecular basis of thymic involution in our patient groups was unclear, we hypothesise that an elevated level of IL-6 is a contributing factor as inflammation drives thymic atrophy.^37^ This state of thymic insufficiency contributes towards a loss of peripheral naïve T cells, and a compensatory increased homoeostatic proliferation and accumulation of central memory and effector memory T cells during early stages of disease. These effector memory T cells differentiate into a terminally differentiated state due to replicative stress; increasingly recognised as a driver of disease pathogenesis.

Cellular senescence is a state of cell cycle arrest in metabolically active cells that secrete high levels of inflammatory cytokines and chemokines, termed the senescence-associated secretory phenotype (SASP); widely accepted as contributors towards inflammation and age-related disease.^38^ An increased frequency of senescent-like CD28^null^ T cells is acknowledged as a key characteristic of immune ageing that has been observed in patients with RA.^39^ We found a 12% higher circulating frequency of senescent T cells only in our patients with established disease irrespective of the symptom duration, suggesting this substantial expansion is not only inflammatory but likely to be pathogenic; making it an attractive intervention target Furthermore, our gene expression analysis revealed increased expression of cellular senescence associated genes [e.g., Cell cycle inhibitor protein CDKNIA] and inflammatory genes [e.g., SERPINB2, HMBG1, CXCL1] that are a part of the recently published gene set that identifies senescent cells and predicts senescence-associated pathways (SenMayo).^19^

CD4 T cells play a key role in RA pathogenesis; particularly Th17 cells, that produce and secrete IL17 which activates other immune cells [such as macrophages, B cells] and promotes migration towards inflammatory sites.^18^ In this study, we observed an expansion of Th17 cells and elevated circulating IL17 levels during the early weeks of disease establishment, possibly a result of the pro-inflammatory environment as cytokines such as IL-6 and IL-23 that has been previously reported.^40^ On the other hand, the compartment of regulatory Foxp3 expressing T cells (Tregs) were only expanded in patients with established RA that may be an attempt to counteract the pro-inflammatory conditions that occur due to an expansion of inflammatory T cells the imbalance between Th17/Treg still persists; thus, contributing towards autoimmunity.^41^

B cells promote RA disease pathogenesis through a variety of mechanisms, including cytokine secretion and autoantibody production. Comparing B cell subgroups in our patient cohorts we identified some features of B cell ageing, including expansion of double negative (CD27^−^IgD) senescent-like B cells that express a SASP, during the early stages prior to disease establishment, suggesting a role in RA pathogenesis.^26^ Others have shown that blocking IL-6 with tocilizumab (an anti-IL-6 receptor (IL-6R) monoclonal antibody) in patients with RA inhibited these senescent-like B cells and improved clinical symptoms, further supporting a pathogenic role for this B cell subset.^42^ We also provide evidence for a role of pathogenic autoantibody secreting ABCs, that express Interferon regulatory factor IRF5 and autoreactive B cells that have differentiated into CD38 expressing plasmablasts, during the pre-clinical disease stage.

The clinical phase of arthralgia that precedes the development of RA is an important window during which early treatment to prevent disease development and progression, but the accurate prediction of RA development is critical to allow interventions to be appropriately target to those at highest risk. ACPA is seen in some, but not all, patients who develop RA, and conversely, some ACPA positive patients with arthralgia never develop arthritis. Limited studies reporting immunological differences in patients with arthralgia that developed arthritis have observed an expansion of activated B cells and CD161 expressing precursor Th17 cells.^43^ Although numbers were small, in this study we have reported a state of heightened immune ageing in the preclinical phase; with the IMM-AGE score higher in ACPA^+ve^ patients with arthralgia that developed RA over a 24 month follow up period. Additionally, an expansion of Age-associated B cells was observed in patients with arthralgia who converted to RA irrespective of ACPA seropositivity. These changes although statistically significant due to the small mean differences they have limited standalone clinical utility in early disease prediction and hints at the utility of a combination of multiple biomarkers [autoantibody levels, markers of immunological ageing to predict which patients with arthralgia will progress to RA.

Our findings are indicative of early appearance of immune ageing features reflected in the higher immunological age in undifferentiated arthritis patients that emerge prior to diseases establishment suggesting that this may contribute to the pathogenesis of RA by reducing immune regulation and facilitating a chronic inflammatory environment. Anti-ageing drugs, targeting central immune-ageing pathways including autophagy dysfunction, impaired clearance of senescent cells and unbalanced metabolism, may thus have potential for preventing treating RA. The anti-diabetic agent metformin targets biological ageing pathways and has effects that are independent of its anti-hyperglycaemic role including, autophagy enhancing and anti-inflammatory effects driven by inhibition of NF-κB signalling. A pilot trials of metformin in patients with RA have demonstrated improvements in disease activity, quality of life and levels of systemic inflammation,^44^ highlighting the potential of metformin as an adjuvant therapy in patients with arthritis possibly driven by a more favourable immunological profile in these patients. Another promising anti-ageing drug is spermidine, a physiological autophagy stimulator,^45^ has been shown to reverse T cell and B cell senescence and boost immune function in aged donors.^46^ In this study, we observed a reduction of autophagy related genes during the early stages of RA. This reduction is critical since enhanced autophagy can reduce the accumulation of damaged proteins and organelles that may be contributing to chronic inflammation. Moreover, spermidine’s anti-inflammatory and anti-immune ageing properties might help mitigate joint inflammation. Notably, in a mouse model of osteoarthritis, administration of spermidine led to an amelioration of cartilage degradation^47^ underscoring its therapeutic potential. Therapeutic approaches targeting pro-inflammatory senescent cells using senolytics has unleashed multiple health benefits in attenuation of age-related phenotypes and extended lifespan in aged mice models.^48^ A senolytics trial in human idiopathic pulmonary fibrosis patients selectively ablating senescent cells using Dasatinib and Quercetin (DQ) alleviates physical dysfunction and circulating SASP factors which have been confirmed in a cohort of inflammatory disease.^49^ Our data provide a rationale for trials of such anti-ageing drugs in patients with arthralgia at risk of developing RA which can be validated in future preclinical and clinical studies to explore dosing and treatment duration to delineate the therapeutic window and maximise therapeutic benefits of proposed geroprotective drugs.

A key limitation of this study is its largely cross-sectional design across different cohorts at various stages of RA development. Whilst the study has provided valuable insights it does not capture the dynamic changes that may occur over time in the same individual as they progress from a healthy state to the onset and then development of RA. Furthermore, an additional limitation is posed due to the sample size in each cohort and further studies with larger and more diverse populations are needed to validate these findings and detailed longitudinal assessment across multiple pre-RA phases would provide a comprehensive understanding of disease development. Additionally, in our study of prognostic indicators only 9 of the patients with arthralgia convert to RA; future studies should explore, in a larger sample, if the IMM-AGE score has utility as a prognostic biomarker in these patients. Due to the collection of a limited volume of blood from the participants, it was not possible to assess immune cell function making it challenging to accurately investigate how the immune ageing related shifts in T and B cell populations impact immune response in RA pathogenesis. Thus, for instance, although we see an expansion of T_regs_ in patients with established of RA, we could not investigate whether these cells were functionally efficient. Furthermore, there was variation in sex distribution amongst our patient groups, and this will have affected the data, though our regression analysis revealed that sex was an influence on the degree of immune ageing only in the undifferentiated arthritis cohort. Finally, despite our efforts to account for known covariates, we cannot exclude the possibility that other unmeasured factors that were not captured in the current dataset may have influenced the observed associations.

## Contributors

**JML, AGP, LP, AVHM, KR** gained funding for the study, participated in the design of the study, interpretation of the data and were involved in drafting the manuscript. **NAD** participated in sample processing, performed the immune phenotyping and transcriptomic analysis, analysed and interpreted the data generated and wrote the first draft of the manuscript. **JML** and **SWJ** have accessed and verified the data reported in the manuscript and were involved in reviewing the manuscript. **KR** and **AF** recruited and followed up the arthritis patients. **ASO** calculated the IMM-AGE scores and did the regression analysis. All authors edited and approved the final version of the manuscript.

## Data sharing statement

The data flow cytometry, cytokine levels and transcriptomics data that support the findings of this study are available on request from the corresponding author (aroran@bham.ac.uk). The data are not publicly available due to ethical restrictions.

## Declaration of interests

AGP has received an in-kind payment from GSK as a part of the EMINENT (GSK/MRC) funding award. The rest of the authors declare that they have no competing interests.

## Appendix A Supplementary data

Supplementary Fig. S1 and Tables S1–S15 Supplementary Fig. S1: CD8 T cell subset distribution across disease phases leading up to the development of Rheumatoid Arthritis. (a) Comparison of the proportion of four CD8 T cell subsets across our 5 cohorts; (b) CD69+ve (c) CD154+ve (d) CD28−veCD57+ve CD8 T cells in PBMCs isolated from healthy age and sex-matched controls (n = 69), patients with arthralgia (n = 32), undifferentiated arthritis (n = 44), early RA whose disease was confirmed in the last 3 months (n = 23) and established RA whose disease was confirmed at longer than 3 months duration (n = 56). Data represent individual values, mean (centre bar). Statistical analysis was done using a One-way ANOVA to demonstrate significant group differences.
