# Supplementary material for: Specific features of immune ageing are detected in the earliest stages in rheumatoid arthritis development
Source: eBioMedicine. 2025 Sep 3;119:105900. doi: 10.1016/j.ebiom.2025.105900 (PMC12789766; doi:10.1016/j.ebiom.2025.105900)
Supplement: Supplementary Fig. S1 and Tables S1–S15 — Supplementary Fig. S1: CD8 T cell subset distribution across disease phases leading up to the development of Rheumatoid Arthritis. (a) Comparison of the proportion of four CD8 T cell subsets across our 5 cohorts; (b) CD69+ve (c) CD154+ve (d) CD28−veCD57+ve CD8 T cells in PBMCs isolated from healthy age and sex-matched controls (n = 69), patients with arthralgia (n = 32), undifferentiated arthritis (n = 44), early RA whose disease was confirmed in the last 3 months (n = 23) and established RA whose disease was confirmed at longer than 3 months duration (n = 56). Data represent individual values, mean (centre bar). Statistical analysis was done using a One-way ANOVA to demonstrate significant group differences. [file mmc1.pptx]

## Slide 1
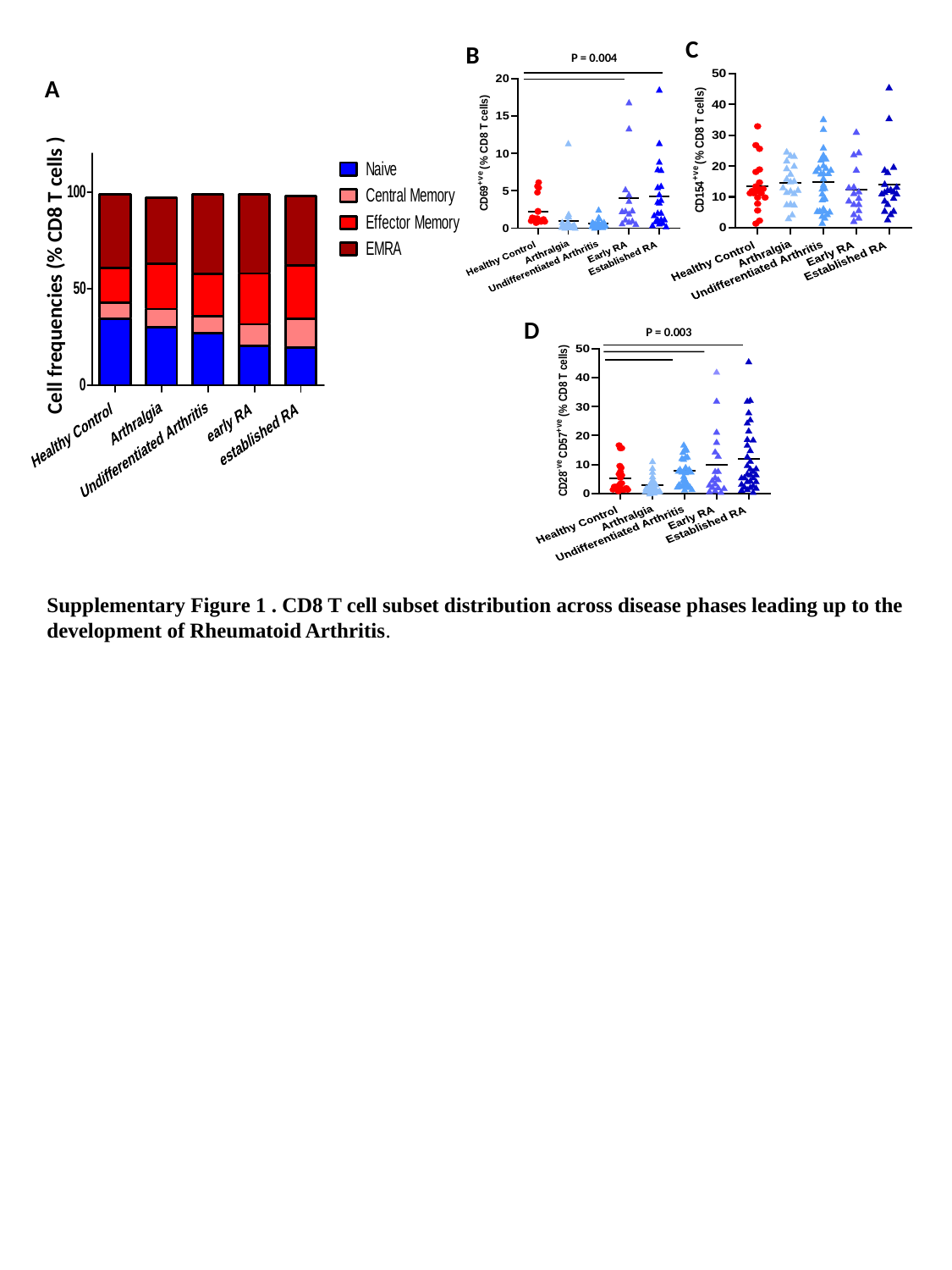

C
B
P = 0.004
A
Cell frequencies (% CD8 T cells )
D
P = 0.003
Supplementary Figure 1 . CD8 T cell subset distribution across disease phases leading up to the development of Rheumatoid Arthritis.

## Slide 2
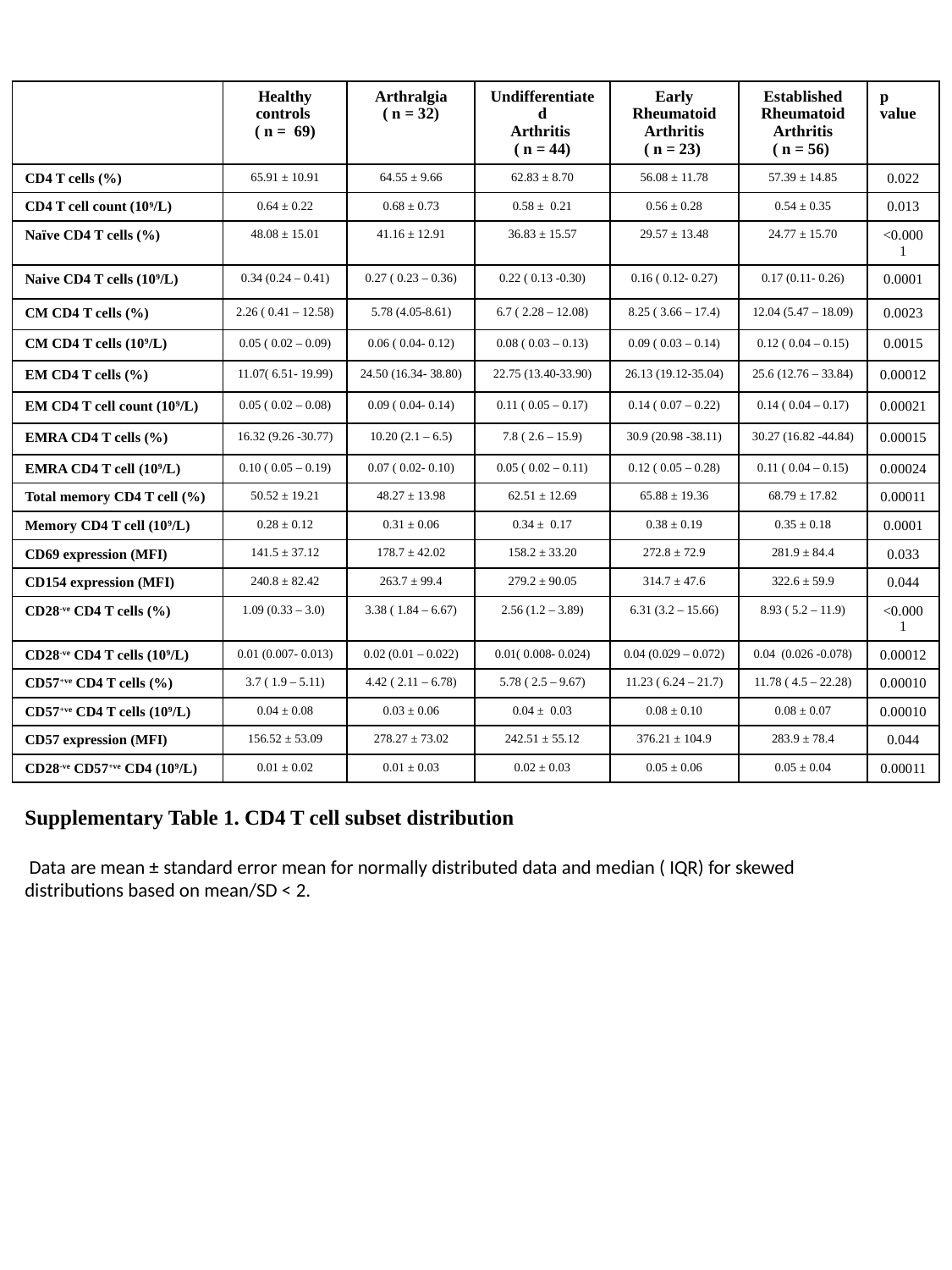

| | Healthy controls ( n = 69) | Arthralgia ( n = 32) | Undifferentiated Arthritis ( n = 44) | Early Rheumatoid Arthritis ( n = 23) | Established Rheumatoid Arthritis ( n = 56) | p value |
| --- | --- | --- | --- | --- | --- | --- |
| CD4 T cells (%) | 65.91 ± 10.91 | 64.55 ± 9.66 | 62.83 ± 8.70 | 56.08 ± 11.78 | 57.39 ± 14.85 | 0.022 |
| CD4 T cell count (109/L) | 0.64 ± 0.22 | 0.68 ± 0.73 | 0.58 ± 0.21 | 0.56 ± 0.28 | 0.54 ± 0.35 | 0.013 |
| Naïve CD4 T cells (%) | 48.08 ± 15.01 | 41.16 ± 12.91 | 36.83 ± 15.57 | 29.57 ± 13.48 | 24.77 ± 15.70 | <0.0001 |
| Naive CD4 T cells (109/L) | 0.34 (0.24 – 0.41) | 0.27 ( 0.23 – 0.36) | 0.22 ( 0.13 -0.30) | 0.16 ( 0.12- 0.27) | 0.17 (0.11- 0.26) | 0.0001 |
| CM CD4 T cells (%) | 2.26 ( 0.41 – 12.58) | 5.78 (4.05-8.61) | 6.7 ( 2.28 – 12.08) | 8.25 ( 3.66 – 17.4) | 12.04 (5.47 – 18.09) | 0.0023 |
| CM CD4 T cells (109/L) | 0.05 ( 0.02 – 0.09) | 0.06 ( 0.04- 0.12) | 0.08 ( 0.03 – 0.13) | 0.09 ( 0.03 – 0.14) | 0.12 ( 0.04 – 0.15) | 0.0015 |
| EM CD4 T cells (%) | 11.07( 6.51- 19.99) | 24.50 (16.34- 38.80) | 22.75 (13.40-33.90) | 26.13 (19.12-35.04) | 25.6 (12.76 – 33.84) | 0.00012 |
| EM CD4 T cell count (109/L) | 0.05 ( 0.02 – 0.08) | 0.09 ( 0.04- 0.14) | 0.11 ( 0.05 – 0.17) | 0.14 ( 0.07 – 0.22) | 0.14 ( 0.04 – 0.17) | 0.00021 |
| EMRA CD4 T cells (%) | 16.32 (9.26 -30.77) | 10.20 (2.1 – 6.5) | 7.8 ( 2.6 – 15.9) | 30.9 (20.98 -38.11) | 30.27 (16.82 -44.84) | 0.00015 |
| EMRA CD4 T cell (109/L) | 0.10 ( 0.05 – 0.19) | 0.07 ( 0.02- 0.10) | 0.05 ( 0.02 – 0.11) | 0.12 ( 0.05 – 0.28) | 0.11 ( 0.04 – 0.15) | 0.00024 |
| Total memory CD4 T cell (%) | 50.52 ± 19.21 | 48.27 ± 13.98 | 62.51 ± 12.69 | 65.88 ± 19.36 | 68.79 ± 17.82 | 0.00011 |
| Memory CD4 T cell (109/L) | 0.28 ± 0.12 | 0.31 ± 0.06 | 0.34 ± 0.17 | 0.38 ± 0.19 | 0.35 ± 0.18 | 0.0001 |
| CD69 expression (MFI) | 141.5 ± 37.12 | 178.7 ± 42.02 | 158.2 ± 33.20 | 272.8 ± 72.9 | 281.9 ± 84.4 | 0.033 |
| CD154 expression (MFI) | 240.8 ± 82.42 | 263.7 ± 99.4 | 279.2 ± 90.05 | 314.7 ± 47.6 | 322.6 ± 59.9 | 0.044 |
| CD28-ve CD4 T cells (%) | 1.09 (0.33 – 3.0) | 3.38 ( 1.84 – 6.67) | 2.56 (1.2 – 3.89) | 6.31 (3.2 – 15.66) | 8.93 ( 5.2 – 11.9) | <0.0001 |
| CD28-ve CD4 T cells (109/L) | 0.01 (0.007- 0.013) | 0.02 (0.01 – 0.022) | 0.01( 0.008- 0.024) | 0.04 (0.029 – 0.072) | 0.04 (0.026 -0.078) | 0.00012 |
| CD57+ve CD4 T cells (%) | 3.7 ( 1.9 – 5.11) | 4.42 ( 2.11 – 6.78) | 5.78 ( 2.5 – 9.67) | 11.23 ( 6.24 – 21.7) | 11.78 ( 4.5 – 22.28) | 0.00010 |
| CD57+ve CD4 T cells (109/L) | 0.04 ± 0.08 | 0.03 ± 0.06 | 0.04 ± 0.03 | 0.08 ± 0.10 | 0.08 ± 0.07 | 0.00010 |
| CD57 expression (MFI) | 156.52 ± 53.09 | 278.27 ± 73.02 | 242.51 ± 55.12 | 376.21 ± 104.9 | 283.9 ± 78.4 | 0.044 |
| CD28-ve CD57+ve CD4 (109/L) | 0.01 ± 0.02 | 0.01 ± 0.03 | 0.02 ± 0.03 | 0.05 ± 0.06 | 0.05 ± 0.04 | 0.00011 |
Supplementary Table 1. CD4 T cell subset distribution
 Data are mean ± standard error mean for normally distributed data and median ( IQR) for skewed distributions based on mean/SD < 2.

## Slide 3
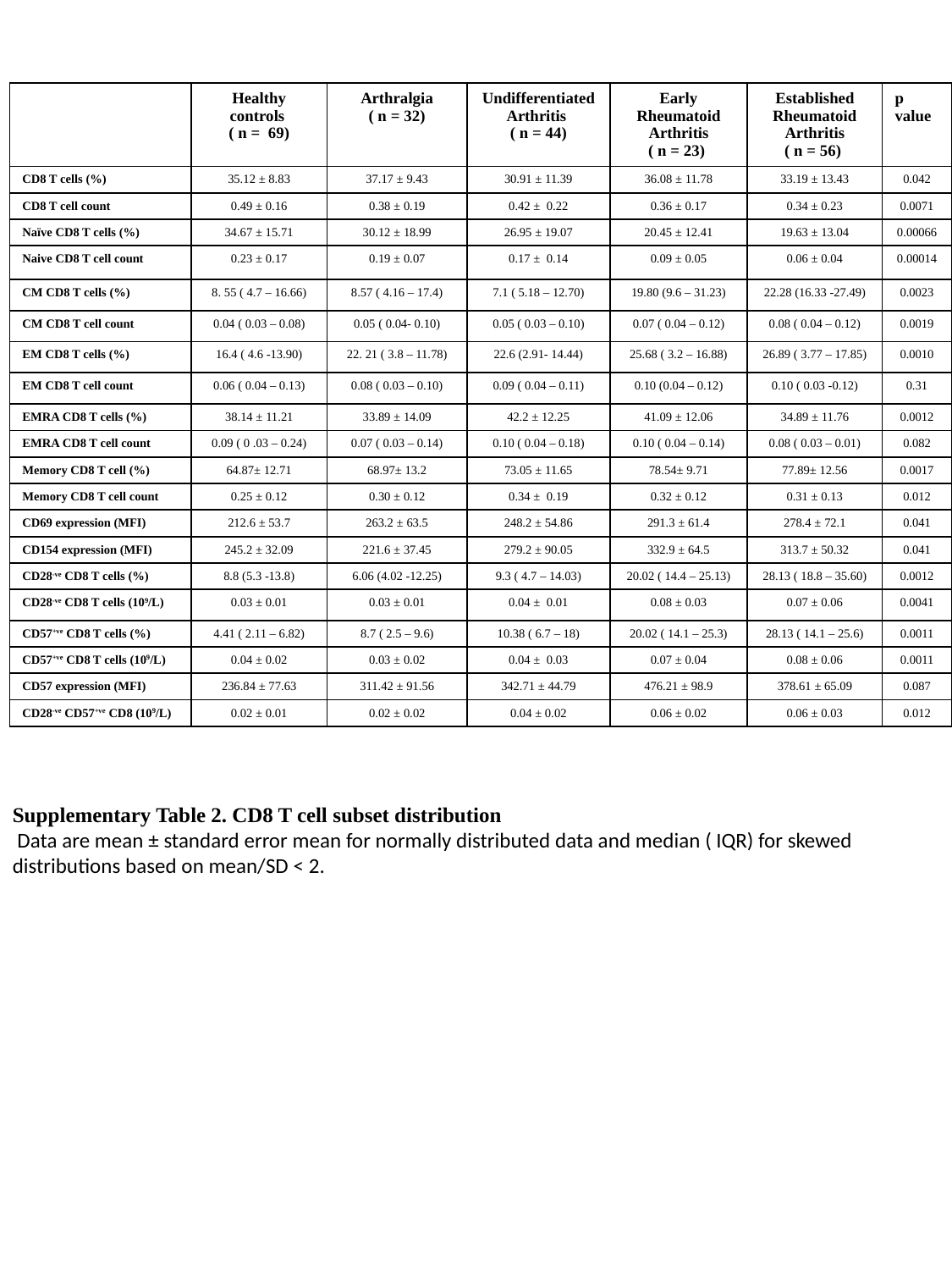

| | Healthy controls ( n = 69) | Arthralgia ( n = 32) | Undifferentiated Arthritis ( n = 44) | Early Rheumatoid Arthritis ( n = 23) | Established Rheumatoid Arthritis ( n = 56) | p value |
| --- | --- | --- | --- | --- | --- | --- |
| CD8 T cells (%) | 35.12 ± 8.83 | 37.17 ± 9.43 | 30.91 ± 11.39 | 36.08 ± 11.78 | 33.19 ± 13.43 | 0.042 |
| CD8 T cell count | 0.49 ± 0.16 | 0.38 ± 0.19 | 0.42 ± 0.22 | 0.36 ± 0.17 | 0.34 ± 0.23 | 0.0071 |
| Naïve CD8 T cells (%) | 34.67 ± 15.71 | 30.12 ± 18.99 | 26.95 ± 19.07 | 20.45 ± 12.41 | 19.63 ± 13.04 | 0.00066 |
| Naive CD8 T cell count | 0.23 ± 0.17 | 0.19 ± 0.07 | 0.17 ± 0.14 | 0.09 ± 0.05 | 0.06 ± 0.04 | 0.00014 |
| CM CD8 T cells (%) | 8. 55 ( 4.7 – 16.66) | 8.57 ( 4.16 – 17.4) | 7.1 ( 5.18 – 12.70) | 19.80 (9.6 – 31.23) | 22.28 (16.33 -27.49) | 0.0023 |
| CM CD8 T cell count | 0.04 ( 0.03 – 0.08) | 0.05 ( 0.04- 0.10) | 0.05 ( 0.03 – 0.10) | 0.07 ( 0.04 – 0.12) | 0.08 ( 0.04 – 0.12) | 0.0019 |
| EM CD8 T cells (%) | 16.4 ( 4.6 -13.90) | 22. 21 ( 3.8 – 11.78) | 22.6 (2.91- 14.44) | 25.68 ( 3.2 – 16.88) | 26.89 ( 3.77 – 17.85) | 0.0010 |
| EM CD8 T cell count | 0.06 ( 0.04 – 0.13) | 0.08 ( 0.03 – 0.10) | 0.09 ( 0.04 – 0.11) | 0.10 (0.04 – 0.12) | 0.10 ( 0.03 -0.12) | 0.31 |
| EMRA CD8 T cells (%) | 38.14 ± 11.21 | 33.89 ± 14.09 | 42.2 ± 12.25 | 41.09 ± 12.06 | 34.89 ± 11.76 | 0.0012 |
| EMRA CD8 T cell count | 0.09 ( 0 .03 – 0.24) | 0.07 ( 0.03 – 0.14) | 0.10 ( 0.04 – 0.18) | 0.10 ( 0.04 – 0.14) | 0.08 ( 0.03 – 0.01) | 0.082 |
| Memory CD8 T cell (%) | 64.87± 12.71 | 68.97± 13.2 | 73.05 ± 11.65 | 78.54± 9.71 | 77.89± 12.56 | 0.0017 |
| Memory CD8 T cell count | 0.25 ± 0.12 | 0.30 ± 0.12 | 0.34 ± 0.19 | 0.32 ± 0.12 | 0.31 ± 0.13 | 0.012 |
| CD69 expression (MFI) | 212.6 ± 53.7 | 263.2 ± 63.5 | 248.2 ± 54.86 | 291.3 ± 61.4 | 278.4 ± 72.1 | 0.041 |
| CD154 expression (MFI) | 245.2 ± 32.09 | 221.6 ± 37.45 | 279.2 ± 90.05 | 332.9 ± 64.5 | 313.7 ± 50.32 | 0.041 |
| CD28-ve CD8 T cells (%) | 8.8 (5.3 -13.8) | 6.06 (4.02 -12.25) | 9.3 ( 4.7 – 14.03) | 20.02 ( 14.4 – 25.13) | 28.13 ( 18.8 – 35.60) | 0.0012 |
| CD28-ve CD8 T cells (109/L) | 0.03 ± 0.01 | 0.03 ± 0.01 | 0.04 ± 0.01 | 0.08 ± 0.03 | 0.07 ± 0.06 | 0.0041 |
| CD57+ve CD8 T cells (%) | 4.41 ( 2.11 – 6.82) | 8.7 ( 2.5 – 9.6) | 10.38 ( 6.7 – 18) | 20.02 ( 14.1 – 25.3) | 28.13 ( 14.1 – 25.6) | 0.0011 |
| CD57+ve CD8 T cells (109/L) | 0.04 ± 0.02 | 0.03 ± 0.02 | 0.04 ± 0.03 | 0.07 ± 0.04 | 0.08 ± 0.06 | 0.0011 |
| CD57 expression (MFI) | 236.84 ± 77.63 | 311.42 ± 91.56 | 342.71 ± 44.79 | 476.21 ± 98.9 | 378.61 ± 65.09 | 0.087 |
| CD28-ve CD57+ve CD8 (109/L) | 0.02 ± 0.01 | 0.02 ± 0.02 | 0.04 ± 0.02 | 0.06 ± 0.02 | 0.06 ± 0.03 | 0.012 |
Supplementary Table 2. CD8 T cell subset distribution
 Data are mean ± standard error mean for normally distributed data and median ( IQR) for skewed distributions based on mean/SD < 2.

## Slide 4
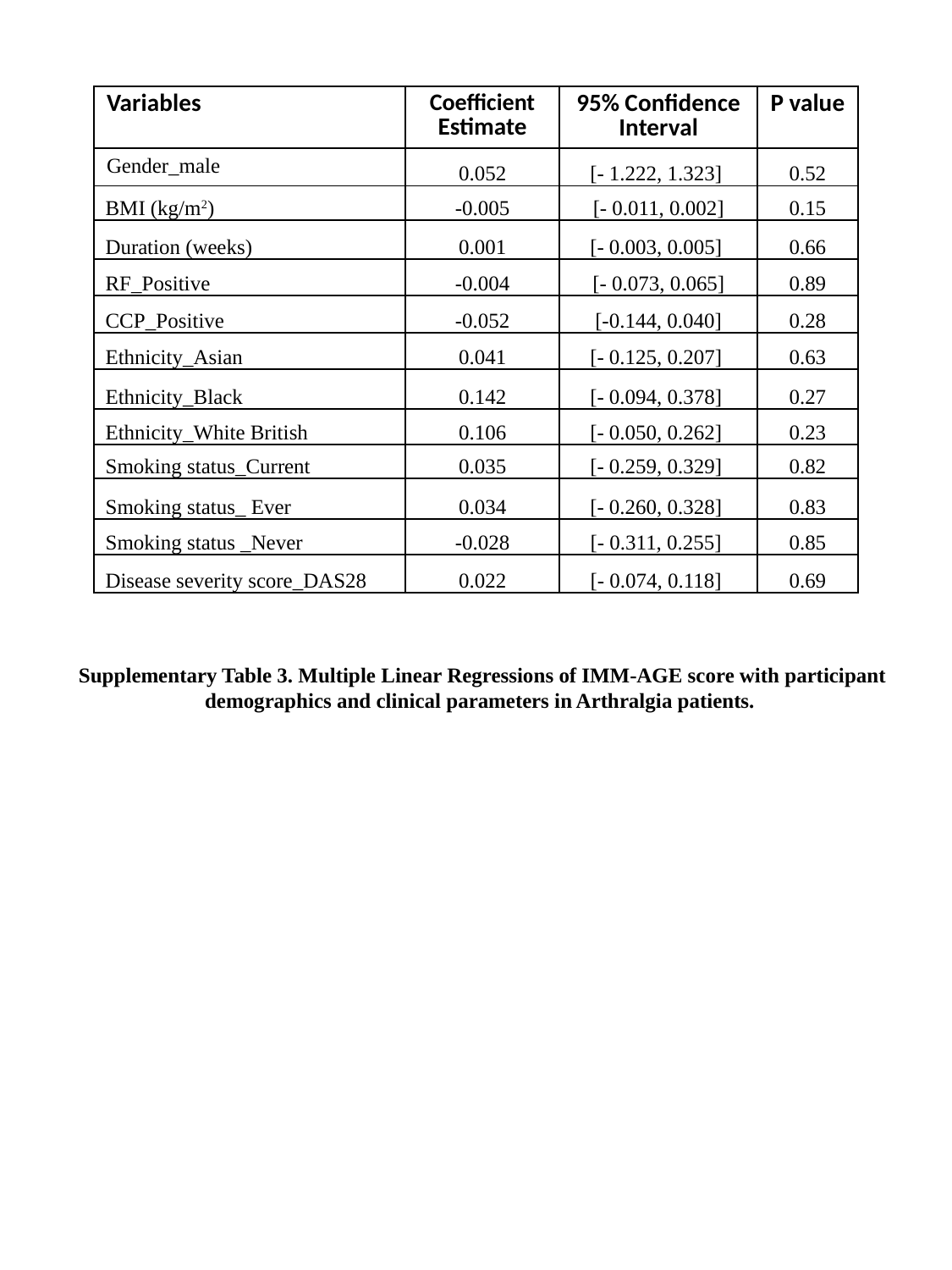

| Variables | Coefficient Estimate | 95% Confidence Interval | P value |
| --- | --- | --- | --- |
| Gender\_male | 0.052 | [- 1.222, 1.323] | 0.52 |
| BMI (kg/m2) | -0.005 | [- 0.011, 0.002] | 0.15 |
| Duration (weeks) | 0.001 | [- 0.003, 0.005] | 0.66 |
| RF\_Positive | -0.004 | [- 0.073, 0.065] | 0.89 |
| CCP\_Positive | -0.052 | [-0.144, 0.040] | 0.28 |
| Ethnicity\_Asian | 0.041 | [- 0.125, 0.207] | 0.63 |
| Ethnicity\_Black | 0.142 | [- 0.094, 0.378] | 0.27 |
| Ethnicity\_White British | 0.106 | [- 0.050, 0.262] | 0.23 |
| Smoking status\_Current | 0.035 | [- 0.259, 0.329] | 0.82 |
| Smoking status\_ Ever | 0.034 | [- 0.260, 0.328] | 0.83 |
| Smoking status \_Never | -0.028 | [- 0.311, 0.255] | 0.85 |
| Disease severity score\_DAS28 | 0.022 | [- 0.074, 0.118] | 0.69 |
Supplementary Table 3. Multiple Linear Regressions of IMM-AGE score with participant demographics and clinical parameters in Arthralgia patients.

## Slide 5
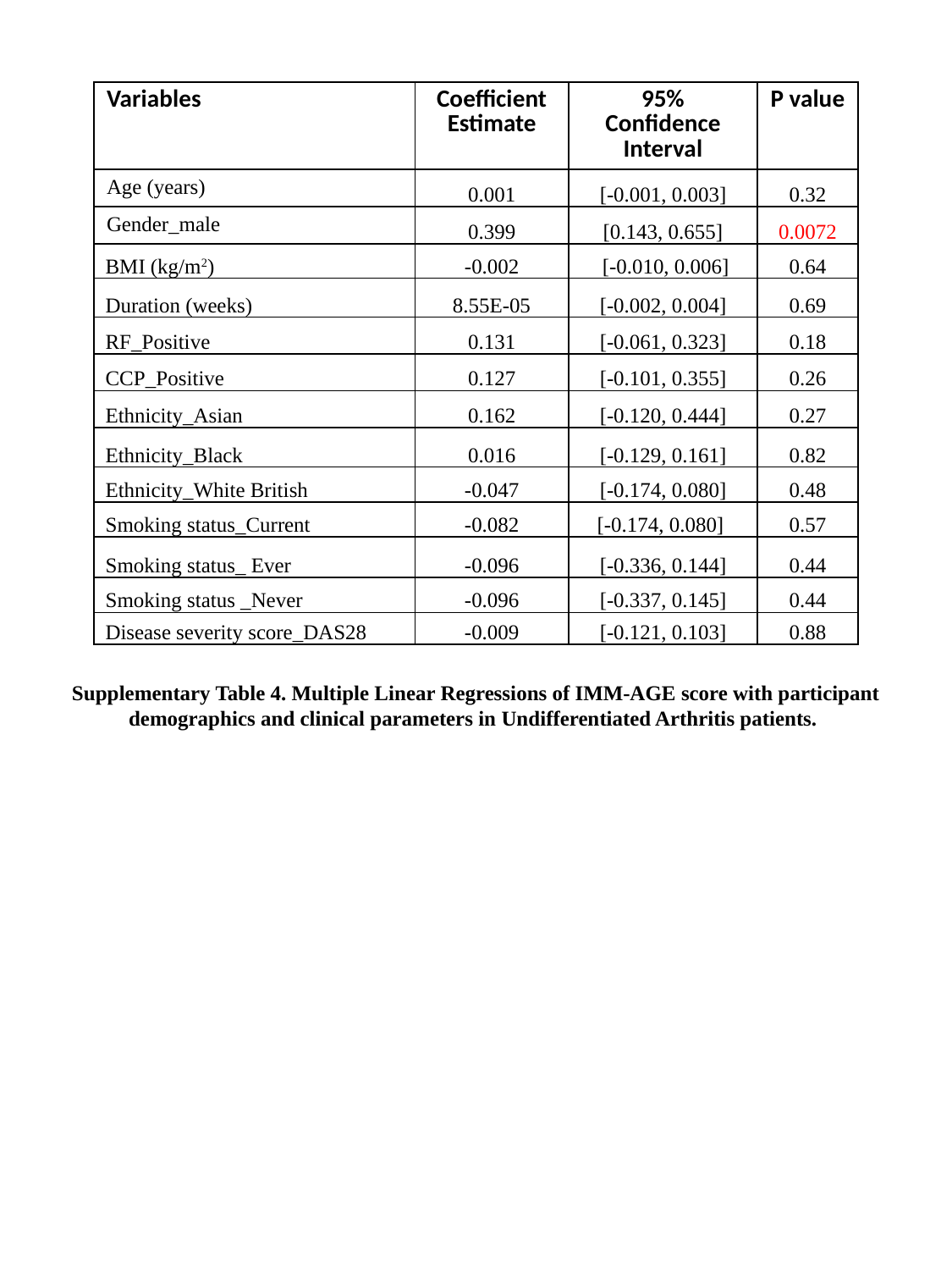

| Variables | Coefficient Estimate | 95% Confidence Interval | P value |
| --- | --- | --- | --- |
| Age (years) | 0.001 | [-0.001, 0.003] | 0.32 |
| Gender\_male | 0.399 | [0.143, 0.655] | 0.0072 |
| BMI (kg/m2) | -0.002 | [-0.010, 0.006] | 0.64 |
| Duration (weeks) | 8.55E-05 | [-0.002, 0.004] | 0.69 |
| RF\_Positive | 0.131 | [-0.061, 0.323] | 0.18 |
| CCP\_Positive | 0.127 | [-0.101, 0.355] | 0.26 |
| Ethnicity\_Asian | 0.162 | [-0.120, 0.444] | 0.27 |
| Ethnicity\_Black | 0.016 | [-0.129, 0.161] | 0.82 |
| Ethnicity\_White British | -0.047 | [-0.174, 0.080] | 0.48 |
| Smoking status\_Current | -0.082 | [-0.174, 0.080] | 0.57 |
| Smoking status\_ Ever | -0.096 | [-0.336, 0.144] | 0.44 |
| Smoking status \_Never | -0.096 | [-0.337, 0.145] | 0.44 |
| Disease severity score\_DAS28 | -0.009 | [-0.121, 0.103] | 0.88 |
Supplementary Table 4. Multiple Linear Regressions of IMM-AGE score with participant demographics and clinical parameters in Undifferentiated Arthritis patients.

## Slide 6
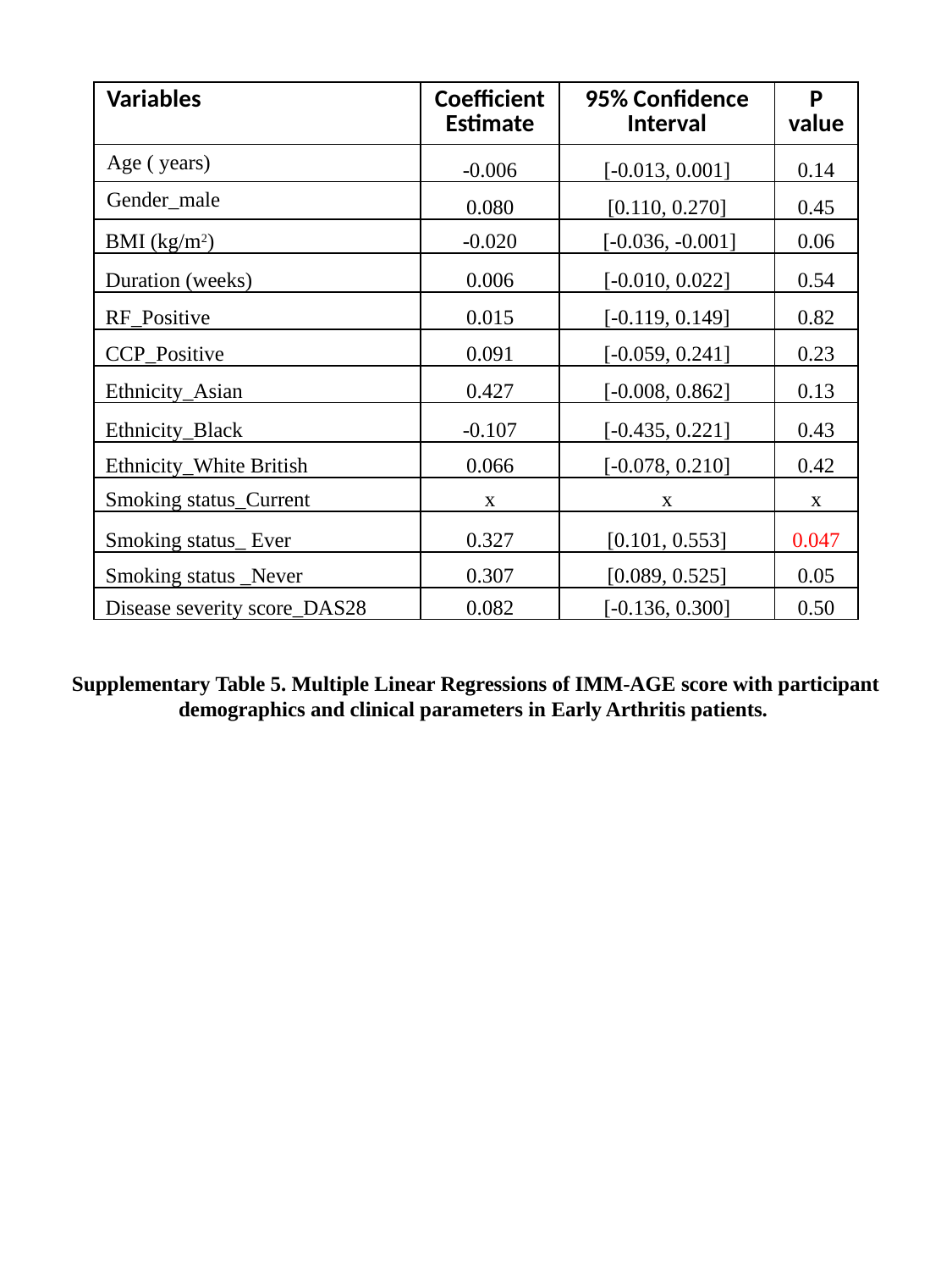

| Variables | Coefficient Estimate | 95% Confidence Interval | P value |
| --- | --- | --- | --- |
| Age ( years) | -0.006 | [-0.013, 0.001] | 0.14 |
| Gender\_male | 0.080 | [0.110, 0.270] | 0.45 |
| BMI (kg/m2) | -0.020 | [-0.036, -0.001] | 0.06 |
| Duration (weeks) | 0.006 | [-0.010, 0.022] | 0.54 |
| RF\_Positive | 0.015 | [-0.119, 0.149] | 0.82 |
| CCP\_Positive | 0.091 | [-0.059, 0.241] | 0.23 |
| Ethnicity\_Asian | 0.427 | [-0.008, 0.862] | 0.13 |
| Ethnicity\_Black | -0.107 | [-0.435, 0.221] | 0.43 |
| Ethnicity\_White British | 0.066 | [-0.078, 0.210] | 0.42 |
| Smoking status\_Current | x | x | x |
| Smoking status\_ Ever | 0.327 | [0.101, 0.553] | 0.047 |
| Smoking status \_Never | 0.307 | [0.089, 0.525] | 0.05 |
| Disease severity score\_DAS28 | 0.082 | [-0.136, 0.300] | 0.50 |
Supplementary Table 5. Multiple Linear Regressions of IMM-AGE score with participant demographics and clinical parameters in Early Arthritis patients.

## Slide 7
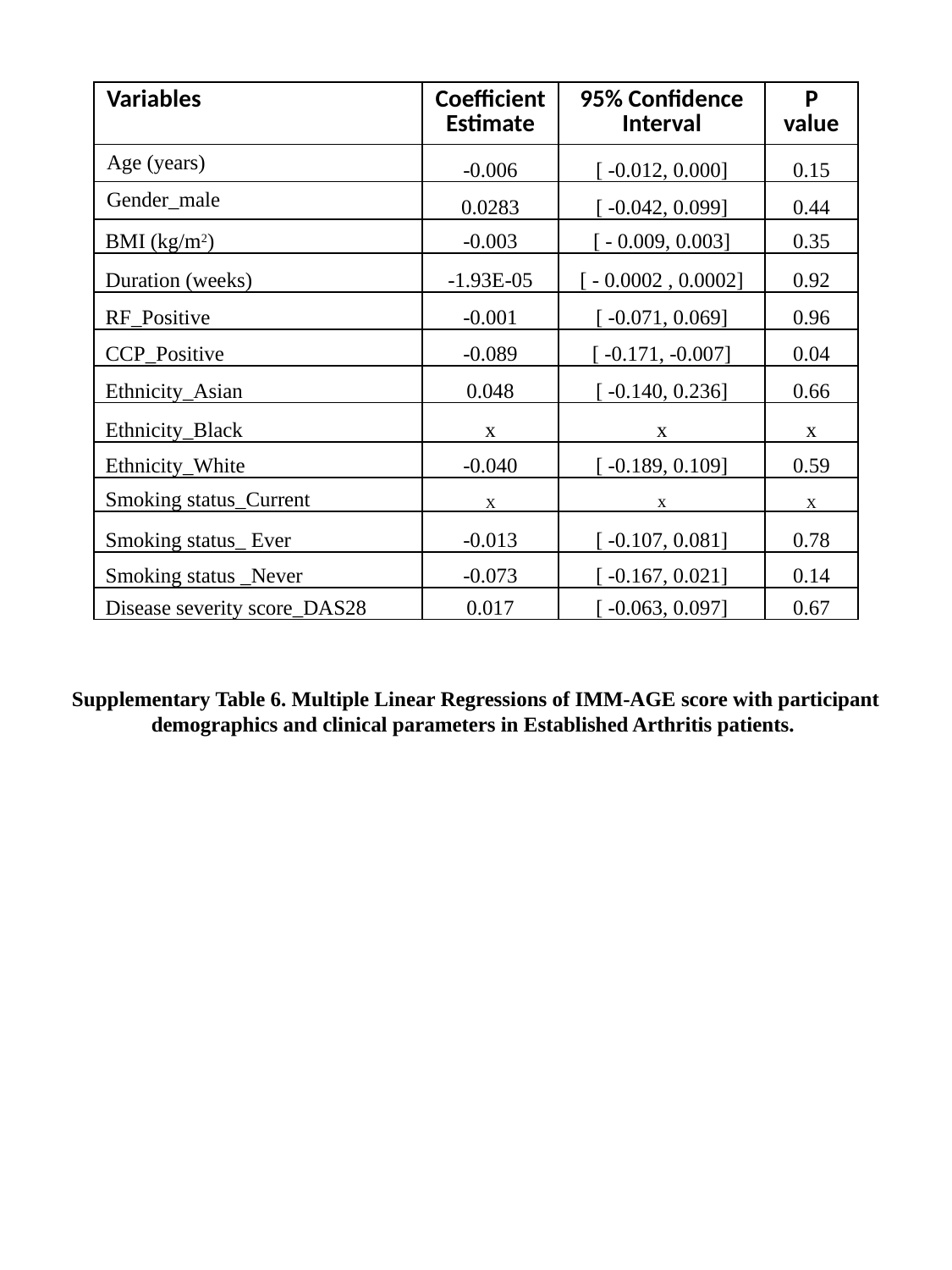

| Variables | Coefficient Estimate | 95% Confidence Interval | P value |
| --- | --- | --- | --- |
| Age (years) | -0.006 | [ -0.012, 0.000] | 0.15 |
| Gender\_male | 0.0283 | [ -0.042, 0.099] | 0.44 |
| BMI (kg/m2) | -0.003 | [ - 0.009, 0.003] | 0.35 |
| Duration (weeks) | -1.93E-05 | [ - 0.0002 , 0.0002] | 0.92 |
| RF\_Positive | -0.001 | [ -0.071, 0.069] | 0.96 |
| CCP\_Positive | -0.089 | [ -0.171, -0.007] | 0.04 |
| Ethnicity\_Asian | 0.048 | [ -0.140, 0.236] | 0.66 |
| Ethnicity\_Black | x | x | x |
| Ethnicity\_White | -0.040 | [ -0.189, 0.109] | 0.59 |
| Smoking status\_Current | X | x | X |
| Smoking status\_ Ever | -0.013 | [ -0.107, 0.081] | 0.78 |
| Smoking status \_Never | -0.073 | [ -0.167, 0.021] | 0.14 |
| Disease severity score\_DAS28 | 0.017 | [ -0.063, 0.097] | 0.67 |
Supplementary Table 6. Multiple Linear Regressions of IMM-AGE score with participant demographics and clinical parameters in Established Arthritis patients.

## Slide 8
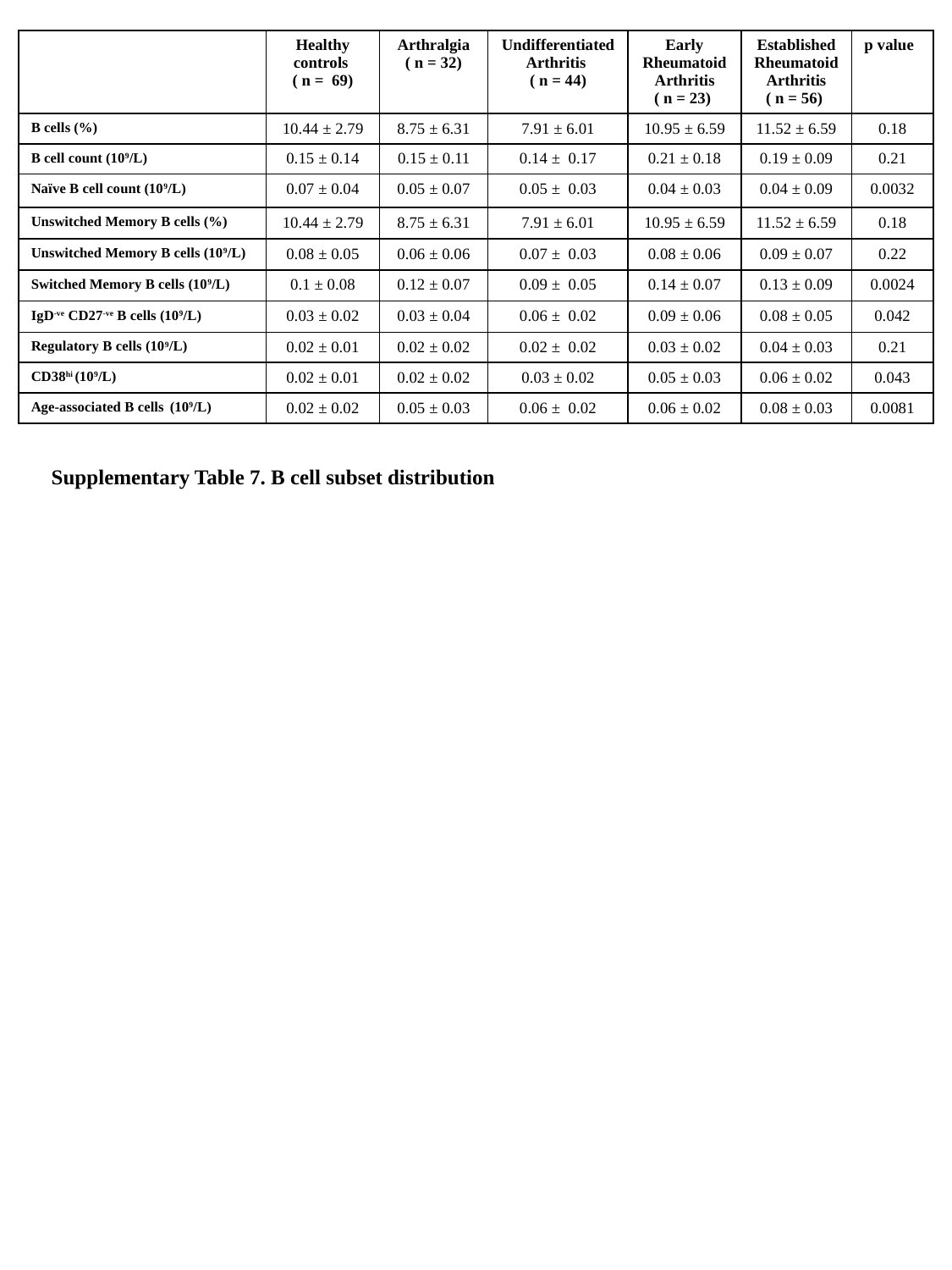

| | Healthy controls ( n = 69) | Arthralgia ( n = 32) | Undifferentiated Arthritis ( n = 44) | Early Rheumatoid Arthritis ( n = 23) | Established Rheumatoid Arthritis ( n = 56) | p value |
| --- | --- | --- | --- | --- | --- | --- |
| B cells (%) | 10.44 ± 2.79 | 8.75 ± 6.31 | 7.91 ± 6.01 | 10.95 ± 6.59 | 11.52 ± 6.59 | 0.18 |
| B cell count (109/L) | 0.15 ± 0.14 | 0.15 ± 0.11 | 0.14 ± 0.17 | 0.21 ± 0.18 | 0.19 ± 0.09 | 0.21 |
| Naïve B cell count (109/L) | 0.07 ± 0.04 | 0.05 ± 0.07 | 0.05 ± 0.03 | 0.04 ± 0.03 | 0.04 ± 0.09 | 0.0032 |
| Unswitched Memory B cells (%) | 10.44 ± 2.79 | 8.75 ± 6.31 | 7.91 ± 6.01 | 10.95 ± 6.59 | 11.52 ± 6.59 | 0.18 |
| Unswitched Memory B cells (109/L) | 0.08 ± 0.05 | 0.06 ± 0.06 | 0.07 ± 0.03 | 0.08 ± 0.06 | 0.09 ± 0.07 | 0.22 |
| Switched Memory B cells (109/L) | 0.1 ± 0.08 | 0.12 ± 0.07 | 0.09 ± 0.05 | 0.14 ± 0.07 | 0.13 ± 0.09 | 0.0024 |
| IgD-ve CD27-ve B cells (109/L) | 0.03 ± 0.02 | 0.03 ± 0.04 | 0.06 ± 0.02 | 0.09 ± 0.06 | 0.08 ± 0.05 | 0.042 |
| Regulatory B cells (109/L) | 0.02 ± 0.01 | 0.02 ± 0.02 | 0.02 ± 0.02 | 0.03 ± 0.02 | 0.04 ± 0.03 | 0.21 |
| CD38hi (109/L) | 0.02 ± 0.01 | 0.02 ± 0.02 | 0.03 ± 0.02 | 0.05 ± 0.03 | 0.06 ± 0.02 | 0.043 |
| Age-associated B cells (109/L) | 0.02 ± 0.02 | 0.05 ± 0.03 | 0.06 ± 0.02 | 0.06 ± 0.02 | 0.08 ± 0.03 | 0.0081 |
Supplementary Table 7. B cell subset distribution

## Slide 9
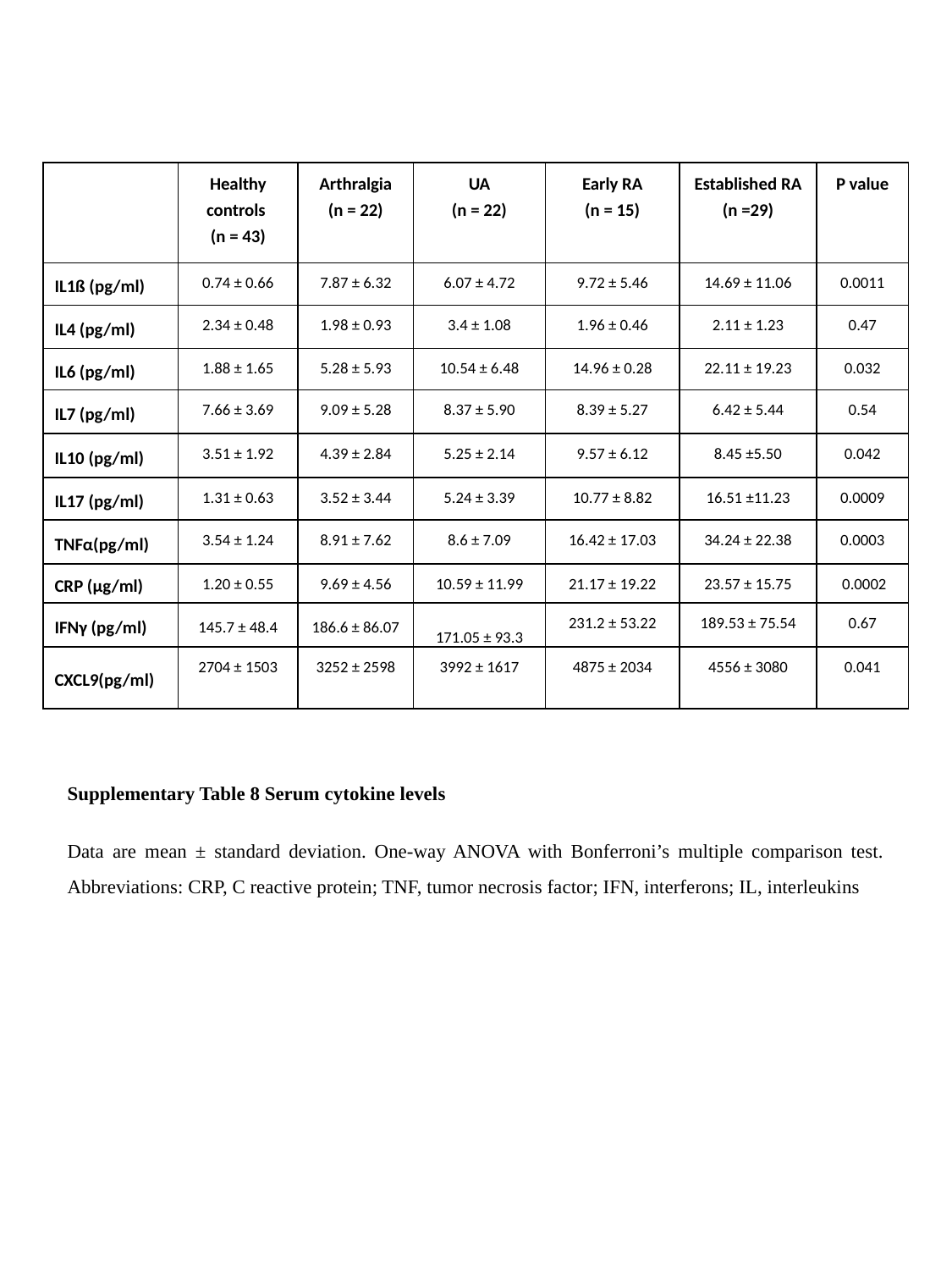

| | Healthy controls (n = 43) | Arthralgia (n = 22) | UA (n = 22) | Early RA (n = 15) | Established RA (n =29) | P value |
| --- | --- | --- | --- | --- | --- | --- |
| IL1ß (pg/ml) | 0.74 ± 0.66 | 7.87 ± 6.32 | 6.07 ± 4.72 | 9.72 ± 5.46 | 14.69 ± 11.06 | 0.0011 |
| IL4 (pg/ml) | 2.34 ± 0.48 | 1.98 ± 0.93 | 3.4 ± 1.08 | 1.96 ± 0.46 | 2.11 ± 1.23 | 0.47 |
| IL6 (pg/ml) | 1.88 ± 1.65 | 5.28 ± 5.93 | 10.54 ± 6.48 | 14.96 ± 0.28 | 22.11 ± 19.23 | 0.032 |
| IL7 (pg/ml) | 7.66 ± 3.69 | 9.09 ± 5.28 | 8.37 ± 5.90 | 8.39 ± 5.27 | 6.42 ± 5.44 | 0.54 |
| IL10 (pg/ml) | 3.51 ± 1.92 | 4.39 ± 2.84 | 5.25 ± 2.14 | 9.57 ± 6.12 | 8.45 ±5.50 | 0.042 |
| IL17 (pg/ml) | 1.31 ± 0.63 | 3.52 ± 3.44 | 5.24 ± 3.39 | 10.77 ± 8.82 | 16.51 ±11.23 | 0.0009 |
| TNFα(pg/ml) | 3.54 ± 1.24 | 8.91 ± 7.62 | 8.6 ± 7.09 | 16.42 ± 17.03 | 34.24 ± 22.38 | 0.0003 |
| CRP (μg/ml) | 1.20 ± 0.55 | 9.69 ± 4.56 | 10.59 ± 11.99 | 21.17 ± 19.22 | 23.57 ± 15.75 | 0.0002 |
| IFNγ (pg/ml) | 145.7 ± 48.4 | 186.6 ± 86.07 | 171.05 ± 93.3 | 231.2 ± 53.22 | 189.53 ± 75.54 | 0.67 |
| CXCL9(pg/ml) | 2704 ± 1503 | 3252 ± 2598 | 3992 ± 1617 | 4875 ± 2034 | 4556 ± 3080 | 0.041 |
Supplementary Table 8 Serum cytokine levels
Data are mean ± standard deviation. One-way ANOVA with Bonferroni’s multiple comparison test. Abbreviations: CRP, C reactive protein; TNF, tumor necrosis factor; IFN, interferons; IL, interleukins

## Slide 10
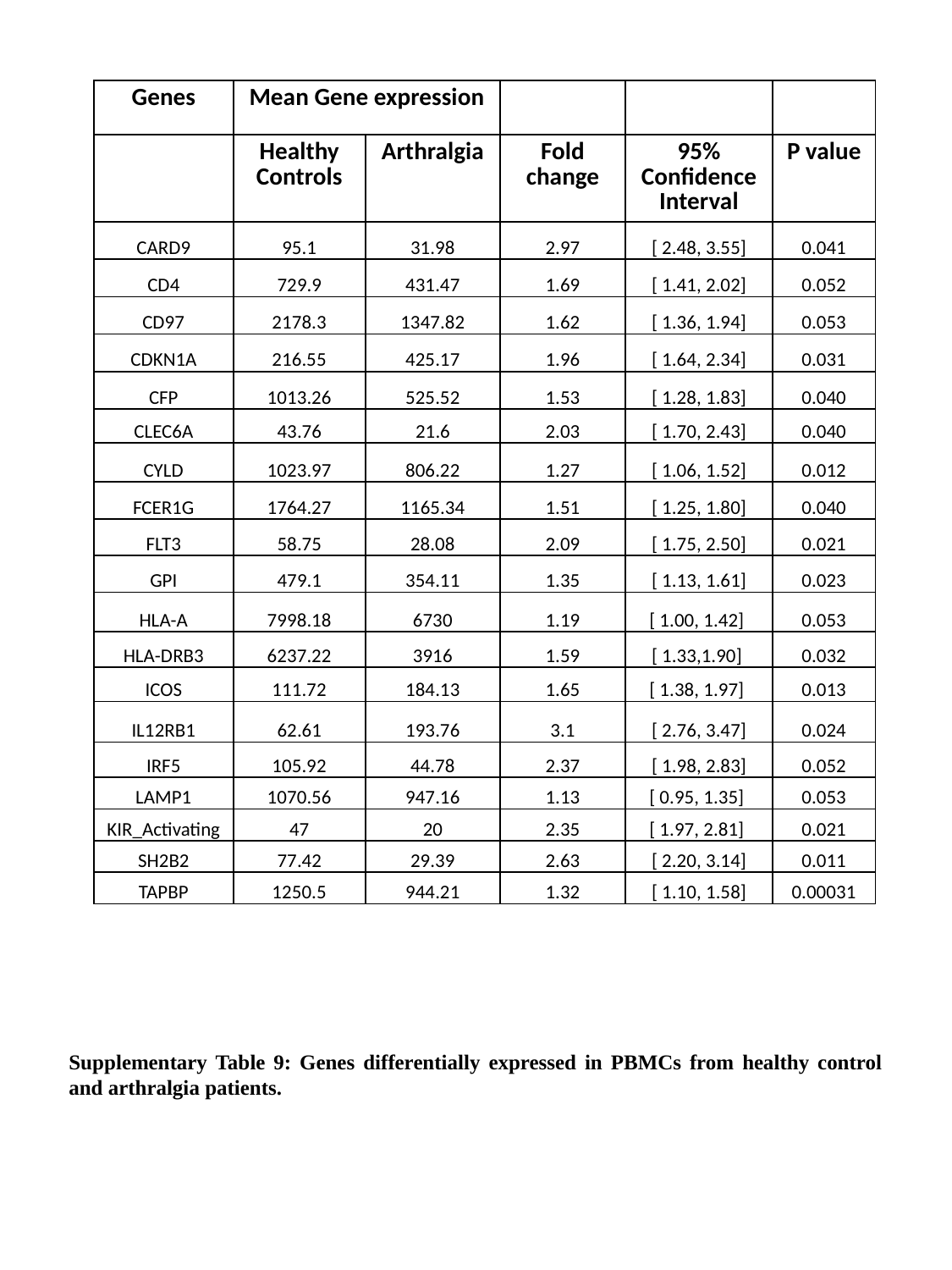

| Genes | Mean Gene expression | | | | |
| --- | --- | --- | --- | --- | --- |
| | Healthy Controls | Arthralgia | Fold change | 95% Confidence Interval | P value |
| CARD9 | 95.1 | 31.98 | 2.97 | [ 2.48, 3.55] | 0.041 |
| CD4 | 729.9 | 431.47 | 1.69 | [ 1.41, 2.02] | 0.052 |
| CD97 | 2178.3 | 1347.82 | 1.62 | [ 1.36, 1.94] | 0.053 |
| CDKN1A | 216.55 | 425.17 | 1.96 | [ 1.64, 2.34] | 0.031 |
| CFP | 1013.26 | 525.52 | 1.53 | [ 1.28, 1.83] | 0.040 |
| CLEC6A | 43.76 | 21.6 | 2.03 | [ 1.70, 2.43] | 0.040 |
| CYLD | 1023.97 | 806.22 | 1.27 | [ 1.06, 1.52] | 0.012 |
| FCER1G | 1764.27 | 1165.34 | 1.51 | [ 1.25, 1.80] | 0.040 |
| FLT3 | 58.75 | 28.08 | 2.09 | [ 1.75, 2.50] | 0.021 |
| GPI | 479.1 | 354.11 | 1.35 | [ 1.13, 1.61] | 0.023 |
| HLA-A | 7998.18 | 6730 | 1.19 | [ 1.00, 1.42] | 0.053 |
| HLA-DRB3 | 6237.22 | 3916 | 1.59 | [ 1.33,1.90] | 0.032 |
| ICOS | 111.72 | 184.13 | 1.65 | [ 1.38, 1.97] | 0.013 |
| IL12RB1 | 62.61 | 193.76 | 3.1 | [ 2.76, 3.47] | 0.024 |
| IRF5 | 105.92 | 44.78 | 2.37 | [ 1.98, 2.83] | 0.052 |
| LAMP1 | 1070.56 | 947.16 | 1.13 | [ 0.95, 1.35] | 0.053 |
| KIR\_Activating | 47 | 20 | 2.35 | [ 1.97, 2.81] | 0.021 |
| SH2B2 | 77.42 | 29.39 | 2.63 | [ 2.20, 3.14] | 0.011 |
| TAPBP | 1250.5 | 944.21 | 1.32 | [ 1.10, 1.58] | 0.00031 |
Supplementary Table 9: Genes differentially expressed in PBMCs from healthy control and arthralgia patients.

## Slide 11
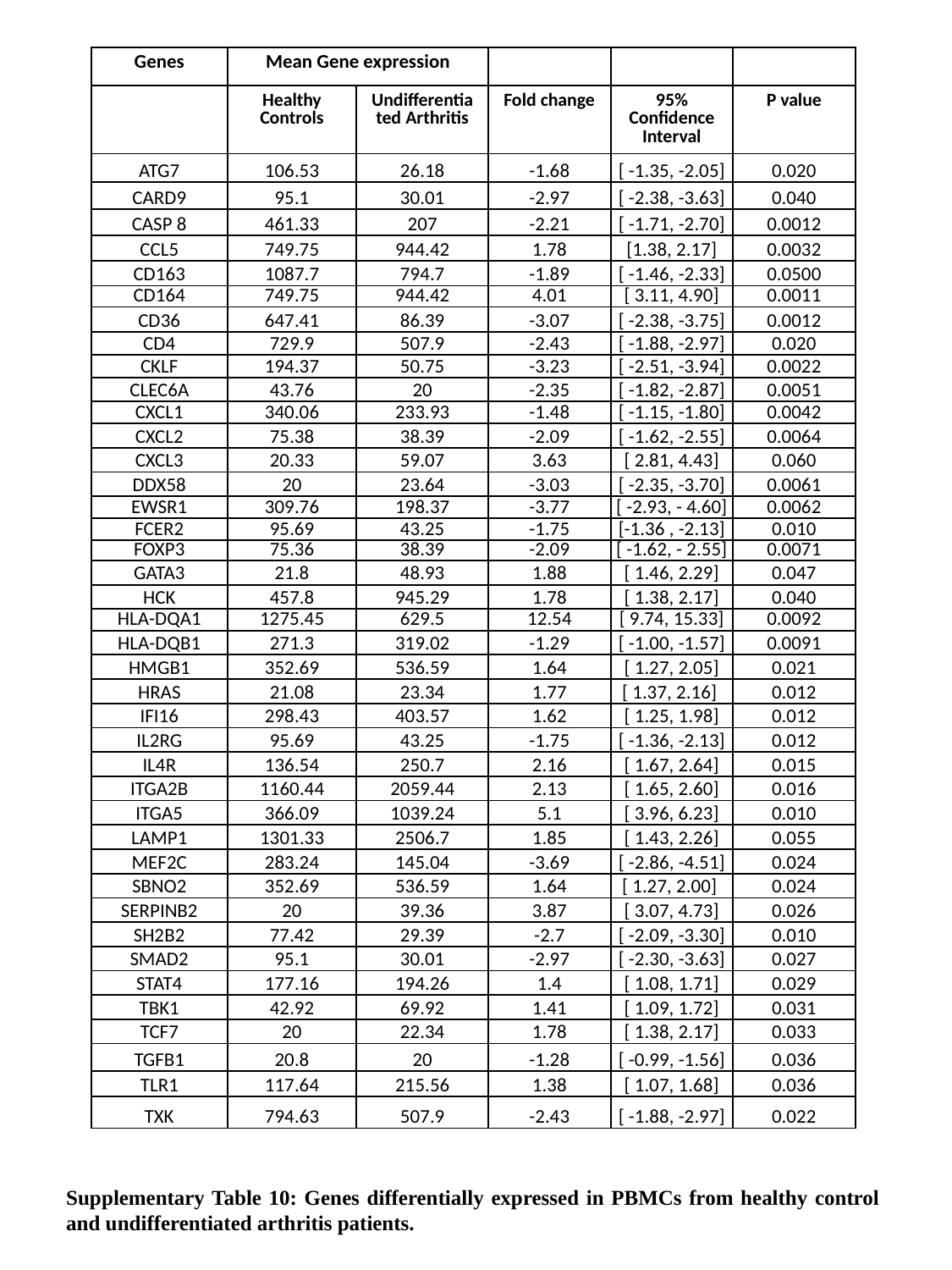

| Genes | Mean Gene expression | | | | |
| --- | --- | --- | --- | --- | --- |
| | Healthy Controls | Undifferentiated Arthritis | Fold change | 95% Confidence Interval | P value |
| ATG7 | 106.53 | 26.18 | -1.68 | [ -1.35, -2.05] | 0.020 |
| CARD9 | 95.1 | 30.01 | -2.97 | [ -2.38, -3.63] | 0.040 |
| CASP 8 | 461.33 | 207 | -2.21 | [ -1.71, -2.70] | 0.0012 |
| CCL5 | 749.75 | 944.42 | 1.78 | [1.38, 2.17] | 0.0032 |
| CD163 | 1087.7 | 794.7 | -1.89 | [ -1.46, -2.33] | 0.0500 |
| CD164 | 749.75 | 944.42 | 4.01 | [ 3.11, 4.90] | 0.0011 |
| CD36 | 647.41 | 86.39 | -3.07 | [ -2.38, -3.75] | 0.0012 |
| CD4 | 729.9 | 507.9 | -2.43 | [ -1.88, -2.97] | 0.020 |
| CKLF | 194.37 | 50.75 | -3.23 | [ -2.51, -3.94] | 0.0022 |
| CLEC6A | 43.76 | 20 | -2.35 | [ -1.82, -2.87] | 0.0051 |
| CXCL1 | 340.06 | 233.93 | -1.48 | [ -1.15, -1.80] | 0.0042 |
| CXCL2 | 75.38 | 38.39 | -2.09 | [ -1.62, -2.55] | 0.0064 |
| CXCL3 | 20.33 | 59.07 | 3.63 | [ 2.81, 4.43] | 0.060 |
| DDX58 | 20 | 23.64 | -3.03 | [ -2.35, -3.70] | 0.0061 |
| EWSR1 | 309.76 | 198.37 | -3.77 | [ -2.93, - 4.60] | 0.0062 |
| FCER2 | 95.69 | 43.25 | -1.75 | [-1.36 , -2.13] | 0.010 |
| FOXP3 | 75.36 | 38.39 | -2.09 | [ -1.62, - 2.55] | 0.0071 |
| GATA3 | 21.8 | 48.93 | 1.88 | [ 1.46, 2.29] | 0.047 |
| HCK | 457.8 | 945.29 | 1.78 | [ 1.38, 2.17] | 0.040 |
| HLA-DQA1 | 1275.45 | 629.5 | 12.54 | [ 9.74, 15.33] | 0.0092 |
| HLA-DQB1 | 271.3 | 319.02 | -1.29 | [ -1.00, -1.57] | 0.0091 |
| HMGB1 | 352.69 | 536.59 | 1.64 | [ 1.27, 2.05] | 0.021 |
| HRAS | 21.08 | 23.34 | 1.77 | [ 1.37, 2.16] | 0.012 |
| IFI16 | 298.43 | 403.57 | 1.62 | [ 1.25, 1.98] | 0.012 |
| IL2RG | 95.69 | 43.25 | -1.75 | [ -1.36, -2.13] | 0.012 |
| IL4R | 136.54 | 250.7 | 2.16 | [ 1.67, 2.64] | 0.015 |
| ITGA2B | 1160.44 | 2059.44 | 2.13 | [ 1.65, 2.60] | 0.016 |
| ITGA5 | 366.09 | 1039.24 | 5.1 | [ 3.96, 6.23] | 0.010 |
| LAMP1 | 1301.33 | 2506.7 | 1.85 | [ 1.43, 2.26] | 0.055 |
| MEF2C | 283.24 | 145.04 | -3.69 | [ -2.86, -4.51] | 0.024 |
| SBNO2 | 352.69 | 536.59 | 1.64 | [ 1.27, 2.00] | 0.024 |
| SERPINB2 | 20 | 39.36 | 3.87 | [ 3.07, 4.73] | 0.026 |
| SH2B2 | 77.42 | 29.39 | -2.7 | [ -2.09, -3.30] | 0.010 |
| SMAD2 | 95.1 | 30.01 | -2.97 | [ -2.30, -3.63] | 0.027 |
| STAT4 | 177.16 | 194.26 | 1.4 | [ 1.08, 1.71] | 0.029 |
| TBK1 | 42.92 | 69.92 | 1.41 | [ 1.09, 1.72] | 0.031 |
| TCF7 | 20 | 22.34 | 1.78 | [ 1.38, 2.17] | 0.033 |
| TGFB1 | 20.8 | 20 | -1.28 | [ -0.99, -1.56] | 0.036 |
| TLR1 | 117.64 | 215.56 | 1.38 | [ 1.07, 1.68] | 0.036 |
| TXK | 794.63 | 507.9 | -2.43 | [ -1.88, -2.97] | 0.022 |
Supplementary Table 10: Genes differentially expressed in PBMCs from healthy control and undifferentiated arthritis patients.

## Slide 12
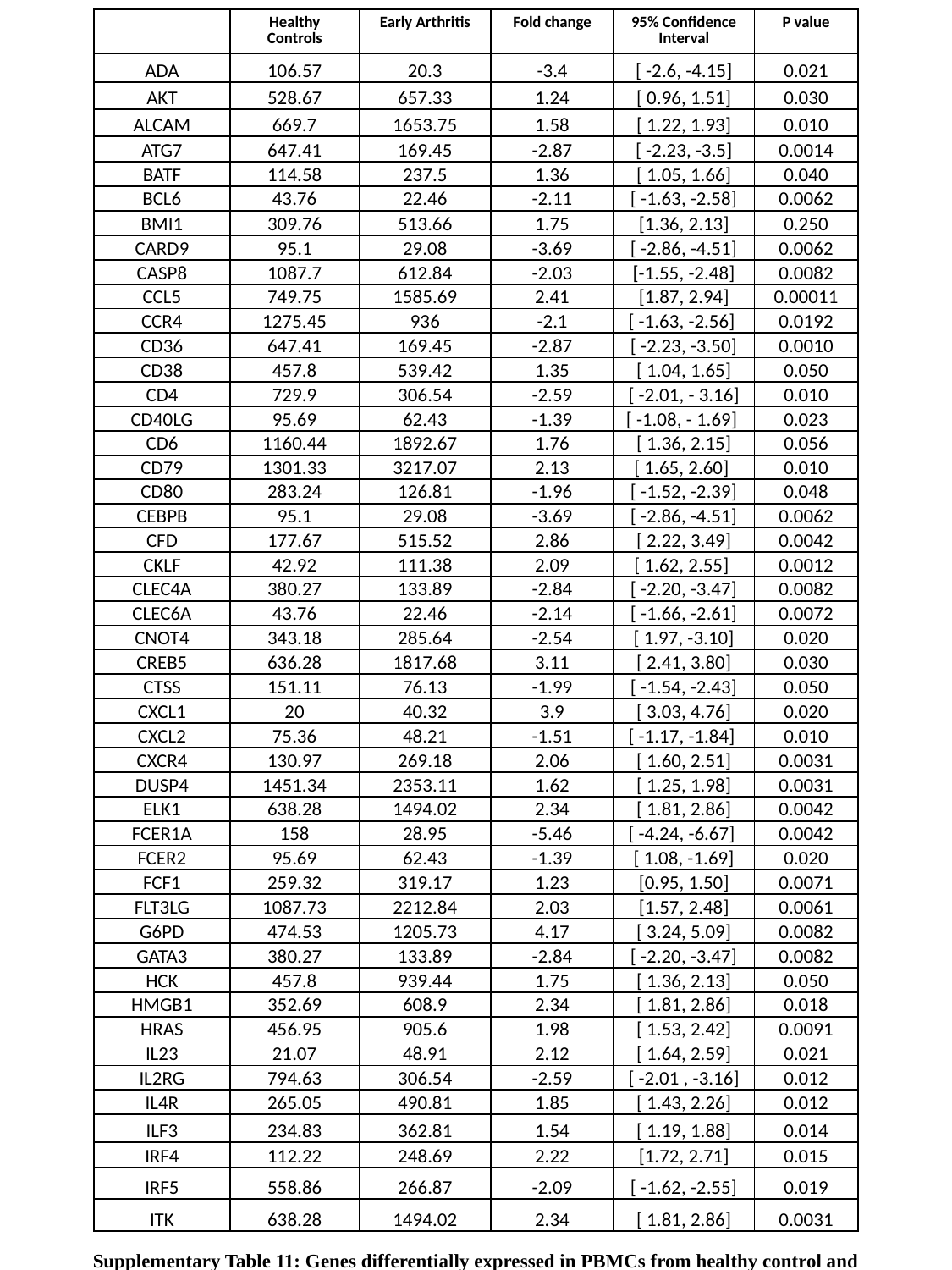

| | Healthy Controls | Early Arthritis | Fold change | 95% Confidence Interval | P value |
| --- | --- | --- | --- | --- | --- |
| ADA | 106.57 | 20.3 | -3.4 | [ -2.6, -4.15] | 0.021 |
| AKT | 528.67 | 657.33 | 1.24 | [ 0.96, 1.51] | 0.030 |
| ALCAM | 669.7 | 1653.75 | 1.58 | [ 1.22, 1.93] | 0.010 |
| ATG7 | 647.41 | 169.45 | -2.87 | [ -2.23, -3.5] | 0.0014 |
| BATF | 114.58 | 237.5 | 1.36 | [ 1.05, 1.66] | 0.040 |
| BCL6 | 43.76 | 22.46 | -2.11 | [ -1.63, -2.58] | 0.0062 |
| BMI1 | 309.76 | 513.66 | 1.75 | [1.36, 2.13] | 0.250 |
| CARD9 | 95.1 | 29.08 | -3.69 | [ -2.86, -4.51] | 0.0062 |
| CASP8 | 1087.7 | 612.84 | -2.03 | [-1.55, -2.48] | 0.0082 |
| CCL5 | 749.75 | 1585.69 | 2.41 | [1.87, 2.94] | 0.00011 |
| CCR4 | 1275.45 | 936 | -2.1 | [ -1.63, -2.56] | 0.0192 |
| CD36 | 647.41 | 169.45 | -2.87 | [ -2.23, -3.50] | 0.0010 |
| CD38 | 457.8 | 539.42 | 1.35 | [ 1.04, 1.65] | 0.050 |
| CD4 | 729.9 | 306.54 | -2.59 | [ -2.01, - 3.16] | 0.010 |
| CD40LG | 95.69 | 62.43 | -1.39 | [ -1.08, - 1.69] | 0.023 |
| CD6 | 1160.44 | 1892.67 | 1.76 | [ 1.36, 2.15] | 0.056 |
| CD79 | 1301.33 | 3217.07 | 2.13 | [ 1.65, 2.60] | 0.010 |
| CD80 | 283.24 | 126.81 | -1.96 | [ -1.52, -2.39] | 0.048 |
| CEBPB | 95.1 | 29.08 | -3.69 | [ -2.86, -4.51] | 0.0062 |
| CFD | 177.67 | 515.52 | 2.86 | [ 2.22, 3.49] | 0.0042 |
| CKLF | 42.92 | 111.38 | 2.09 | [ 1.62, 2.55] | 0.0012 |
| CLEC4A | 380.27 | 133.89 | -2.84 | [ -2.20, -3.47] | 0.0082 |
| CLEC6A | 43.76 | 22.46 | -2.14 | [ -1.66, -2.61] | 0.0072 |
| CNOT4 | 343.18 | 285.64 | -2.54 | [ 1.97, -3.10] | 0.020 |
| CREB5 | 636.28 | 1817.68 | 3.11 | [ 2.41, 3.80] | 0.030 |
| CTSS | 151.11 | 76.13 | -1.99 | [ -1.54, -2.43] | 0.050 |
| CXCL1 | 20 | 40.32 | 3.9 | [ 3.03, 4.76] | 0.020 |
| CXCL2 | 75.36 | 48.21 | -1.51 | [ -1.17, -1.84] | 0.010 |
| CXCR4 | 130.97 | 269.18 | 2.06 | [ 1.60, 2.51] | 0.0031 |
| DUSP4 | 1451.34 | 2353.11 | 1.62 | [ 1.25, 1.98] | 0.0031 |
| ELK1 | 638.28 | 1494.02 | 2.34 | [ 1.81, 2.86] | 0.0042 |
| FCER1A | 158 | 28.95 | -5.46 | [ -4.24, -6.67] | 0.0042 |
| FCER2 | 95.69 | 62.43 | -1.39 | [ 1.08, -1.69] | 0.020 |
| FCF1 | 259.32 | 319.17 | 1.23 | [0.95, 1.50] | 0.0071 |
| FLT3LG | 1087.73 | 2212.84 | 2.03 | [1.57, 2.48] | 0.0061 |
| G6PD | 474.53 | 1205.73 | 4.17 | [ 3.24, 5.09] | 0.0082 |
| GATA3 | 380.27 | 133.89 | -2.84 | [ -2.20, -3.47] | 0.0082 |
| HCK | 457.8 | 939.44 | 1.75 | [ 1.36, 2.13] | 0.050 |
| HMGB1 | 352.69 | 608.9 | 2.34 | [ 1.81, 2.86] | 0.018 |
| HRAS | 456.95 | 905.6 | 1.98 | [ 1.53, 2.42] | 0.0091 |
| IL23 | 21.07 | 48.91 | 2.12 | [ 1.64, 2.59] | 0.021 |
| IL2RG | 794.63 | 306.54 | -2.59 | [ -2.01 , -3.16] | 0.012 |
| IL4R | 265.05 | 490.81 | 1.85 | [ 1.43, 2.26] | 0.012 |
| ILF3 | 234.83 | 362.81 | 1.54 | [ 1.19, 1.88] | 0.014 |
| IRF4 | 112.22 | 248.69 | 2.22 | [1.72, 2.71] | 0.015 |
| IRF5 | 558.86 | 266.87 | -2.09 | [ -1.62, -2.55] | 0.019 |
| ITK | 638.28 | 1494.02 | 2.34 | [ 1.81, 2.86] | 0.0031 |
Supplementary Table 11: Genes differentially expressed in PBMCs from healthy control and Early arthritis patients.

## Slide 13
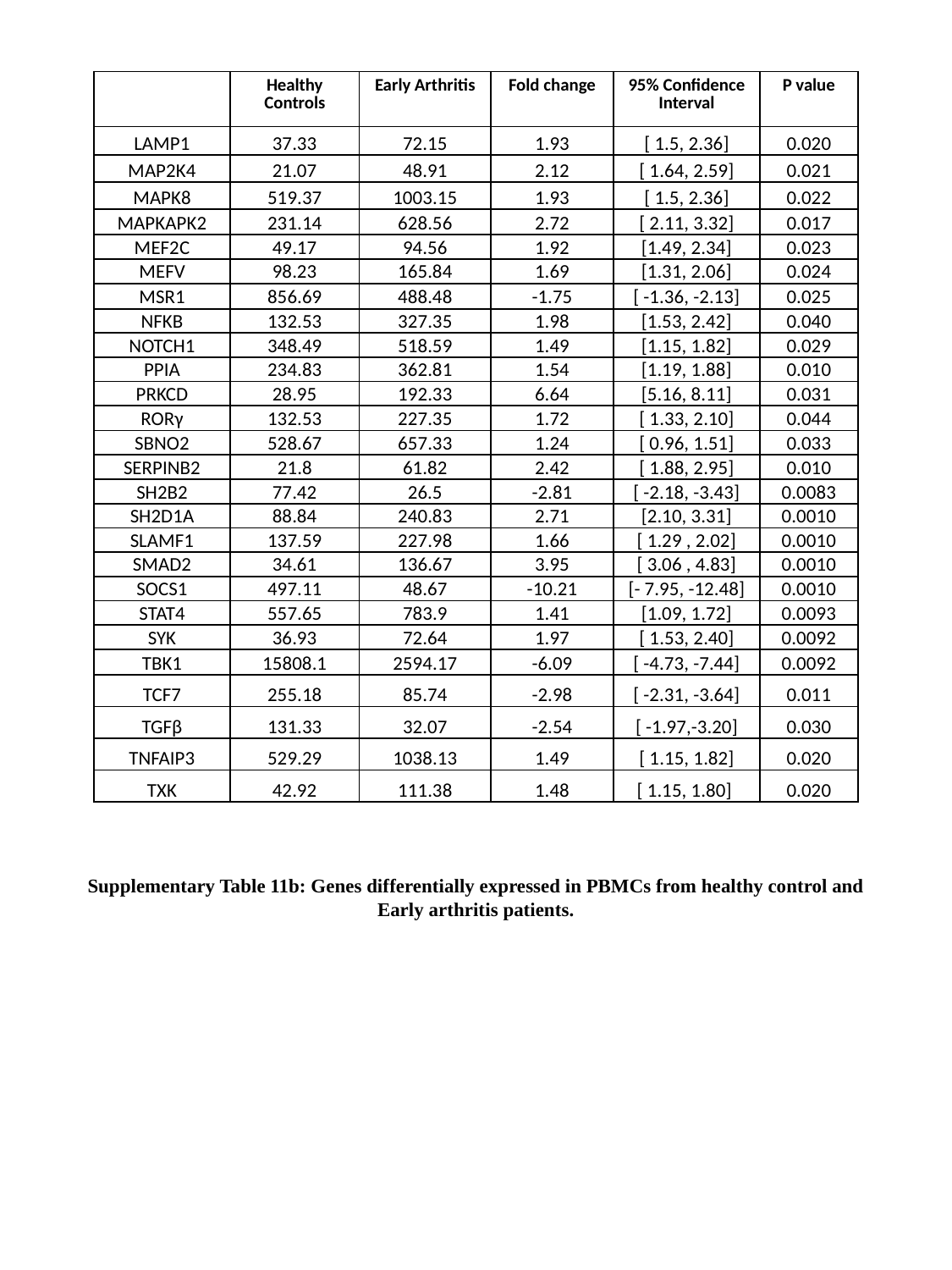

| | Healthy Controls | Early Arthritis | Fold change | 95% Confidence Interval | P value |
| --- | --- | --- | --- | --- | --- |
| LAMP1 | 37.33 | 72.15 | 1.93 | [ 1.5, 2.36] | 0.020 |
| MAP2K4 | 21.07 | 48.91 | 2.12 | [ 1.64, 2.59] | 0.021 |
| MAPK8 | 519.37 | 1003.15 | 1.93 | [ 1.5, 2.36] | 0.022 |
| MAPKAPK2 | 231.14 | 628.56 | 2.72 | [ 2.11, 3.32] | 0.017 |
| MEF2C | 49.17 | 94.56 | 1.92 | [1.49, 2.34] | 0.023 |
| MEFV | 98.23 | 165.84 | 1.69 | [1.31, 2.06] | 0.024 |
| MSR1 | 856.69 | 488.48 | -1.75 | [ -1.36, -2.13] | 0.025 |
| NFKB | 132.53 | 327.35 | 1.98 | [1.53, 2.42] | 0.040 |
| NOTCH1 | 348.49 | 518.59 | 1.49 | [1.15, 1.82] | 0.029 |
| PPIA | 234.83 | 362.81 | 1.54 | [1.19, 1.88] | 0.010 |
| PRKCD | 28.95 | 192.33 | 6.64 | [5.16, 8.11] | 0.031 |
| RORγ | 132.53 | 227.35 | 1.72 | [ 1.33, 2.10] | 0.044 |
| SBNO2 | 528.67 | 657.33 | 1.24 | [ 0.96, 1.51] | 0.033 |
| SERPINB2 | 21.8 | 61.82 | 2.42 | [ 1.88, 2.95] | 0.010 |
| SH2B2 | 77.42 | 26.5 | -2.81 | [ -2.18, -3.43] | 0.0083 |
| SH2D1A | 88.84 | 240.83 | 2.71 | [2.10, 3.31] | 0.0010 |
| SLAMF1 | 137.59 | 227.98 | 1.66 | [ 1.29 , 2.02] | 0.0010 |
| SMAD2 | 34.61 | 136.67 | 3.95 | [ 3.06 , 4.83] | 0.0010 |
| SOCS1 | 497.11 | 48.67 | -10.21 | [- 7.95, -12.48] | 0.0010 |
| STAT4 | 557.65 | 783.9 | 1.41 | [1.09, 1.72] | 0.0093 |
| SYK | 36.93 | 72.64 | 1.97 | [ 1.53, 2.40] | 0.0092 |
| TBK1 | 15808.1 | 2594.17 | -6.09 | [ -4.73, -7.44] | 0.0092 |
| TCF7 | 255.18 | 85.74 | -2.98 | [ -2.31, -3.64] | 0.011 |
| TGFβ | 131.33 | 32.07 | -2.54 | [ -1.97,-3.20] | 0.030 |
| TNFAIP3 | 529.29 | 1038.13 | 1.49 | [ 1.15, 1.82] | 0.020 |
| TXK | 42.92 | 111.38 | 1.48 | [ 1.15, 1.80] | 0.020 |
Supplementary Table 11b: Genes differentially expressed in PBMCs from healthy control and Early arthritis patients.

## Slide 14
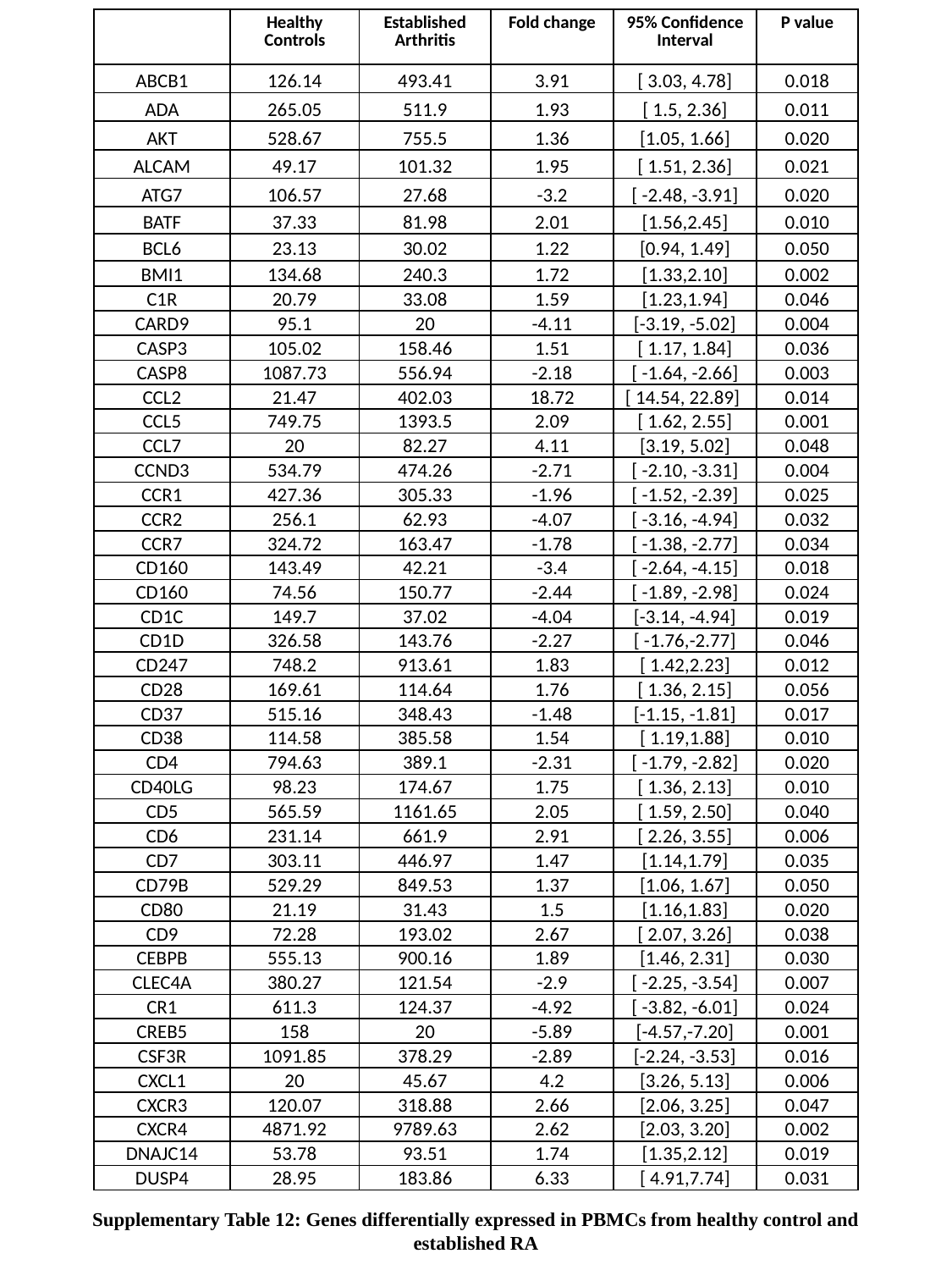

| | Healthy Controls | Established Arthritis | Fold change | 95% Confidence Interval | P value |
| --- | --- | --- | --- | --- | --- |
| ABCB1 | 126.14 | 493.41 | 3.91 | [ 3.03, 4.78] | 0.018 |
| ADA | 265.05 | 511.9 | 1.93 | [ 1.5, 2.36] | 0.011 |
| AKT | 528.67 | 755.5 | 1.36 | [1.05, 1.66] | 0.020 |
| ALCAM | 49.17 | 101.32 | 1.95 | [ 1.51, 2.36] | 0.021 |
| ATG7 | 106.57 | 27.68 | -3.2 | [ -2.48, -3.91] | 0.020 |
| BATF | 37.33 | 81.98 | 2.01 | [1.56,2.45] | 0.010 |
| BCL6 | 23.13 | 30.02 | 1.22 | [0.94, 1.49] | 0.050 |
| BMI1 | 134.68 | 240.3 | 1.72 | [1.33,2.10] | 0.002 |
| C1R | 20.79 | 33.08 | 1.59 | [1.23,1.94] | 0.046 |
| CARD9 | 95.1 | 20 | -4.11 | [-3.19, -5.02] | 0.004 |
| CASP3 | 105.02 | 158.46 | 1.51 | [ 1.17, 1.84] | 0.036 |
| CASP8 | 1087.73 | 556.94 | -2.18 | [ -1.64, -2.66] | 0.003 |
| CCL2 | 21.47 | 402.03 | 18.72 | [ 14.54, 22.89] | 0.014 |
| CCL5 | 749.75 | 1393.5 | 2.09 | [ 1.62, 2.55] | 0.001 |
| CCL7 | 20 | 82.27 | 4.11 | [3.19, 5.02] | 0.048 |
| CCND3 | 534.79 | 474.26 | -2.71 | [ -2.10, -3.31] | 0.004 |
| CCR1 | 427.36 | 305.33 | -1.96 | [ -1.52, -2.39] | 0.025 |
| CCR2 | 256.1 | 62.93 | -4.07 | [ -3.16, -4.94] | 0.032 |
| CCR7 | 324.72 | 163.47 | -1.78 | [ -1.38, -2.77] | 0.034 |
| CD160 | 143.49 | 42.21 | -3.4 | [ -2.64, -4.15] | 0.018 |
| CD160 | 74.56 | 150.77 | -2.44 | [ -1.89, -2.98] | 0.024 |
| CD1C | 149.7 | 37.02 | -4.04 | [-3.14, -4.94] | 0.019 |
| CD1D | 326.58 | 143.76 | -2.27 | [ -1.76,-2.77] | 0.046 |
| CD247 | 748.2 | 913.61 | 1.83 | [ 1.42,2.23] | 0.012 |
| CD28 | 169.61 | 114.64 | 1.76 | [ 1.36, 2.15] | 0.056 |
| CD37 | 515.16 | 348.43 | -1.48 | [-1.15, -1.81] | 0.017 |
| CD38 | 114.58 | 385.58 | 1.54 | [ 1.19,1.88] | 0.010 |
| CD4 | 794.63 | 389.1 | -2.31 | [ -1.79, -2.82] | 0.020 |
| CD40LG | 98.23 | 174.67 | 1.75 | [ 1.36, 2.13] | 0.010 |
| CD5 | 565.59 | 1161.65 | 2.05 | [ 1.59, 2.50] | 0.040 |
| CD6 | 231.14 | 661.9 | 2.91 | [ 2.26, 3.55] | 0.006 |
| CD7 | 303.11 | 446.97 | 1.47 | [1.14,1.79] | 0.035 |
| CD79B | 529.29 | 849.53 | 1.37 | [1.06, 1.67] | 0.050 |
| CD80 | 21.19 | 31.43 | 1.5 | [1.16,1.83] | 0.020 |
| CD9 | 72.28 | 193.02 | 2.67 | [ 2.07, 3.26] | 0.038 |
| CEBPB | 555.13 | 900.16 | 1.89 | [1.46, 2.31] | 0.030 |
| CLEC4A | 380.27 | 121.54 | -2.9 | [ -2.25, -3.54] | 0.007 |
| CR1 | 611.3 | 124.37 | -4.92 | [ -3.82, -6.01] | 0.024 |
| CREB5 | 158 | 20 | -5.89 | [-4.57,-7.20] | 0.001 |
| CSF3R | 1091.85 | 378.29 | -2.89 | [-2.24, -3.53] | 0.016 |
| CXCL1 | 20 | 45.67 | 4.2 | [3.26, 5.13] | 0.006 |
| CXCR3 | 120.07 | 318.88 | 2.66 | [2.06, 3.25] | 0.047 |
| CXCR4 | 4871.92 | 9789.63 | 2.62 | [2.03, 3.20] | 0.002 |
| DNAJC14 | 53.78 | 93.51 | 1.74 | [1.35,2.12] | 0.019 |
| DUSP4 | 28.95 | 183.86 | 6.33 | [ 4.91,7.74] | 0.031 |
Supplementary Table 12: Genes differentially expressed in PBMCs from healthy control and established RA

## Slide 15
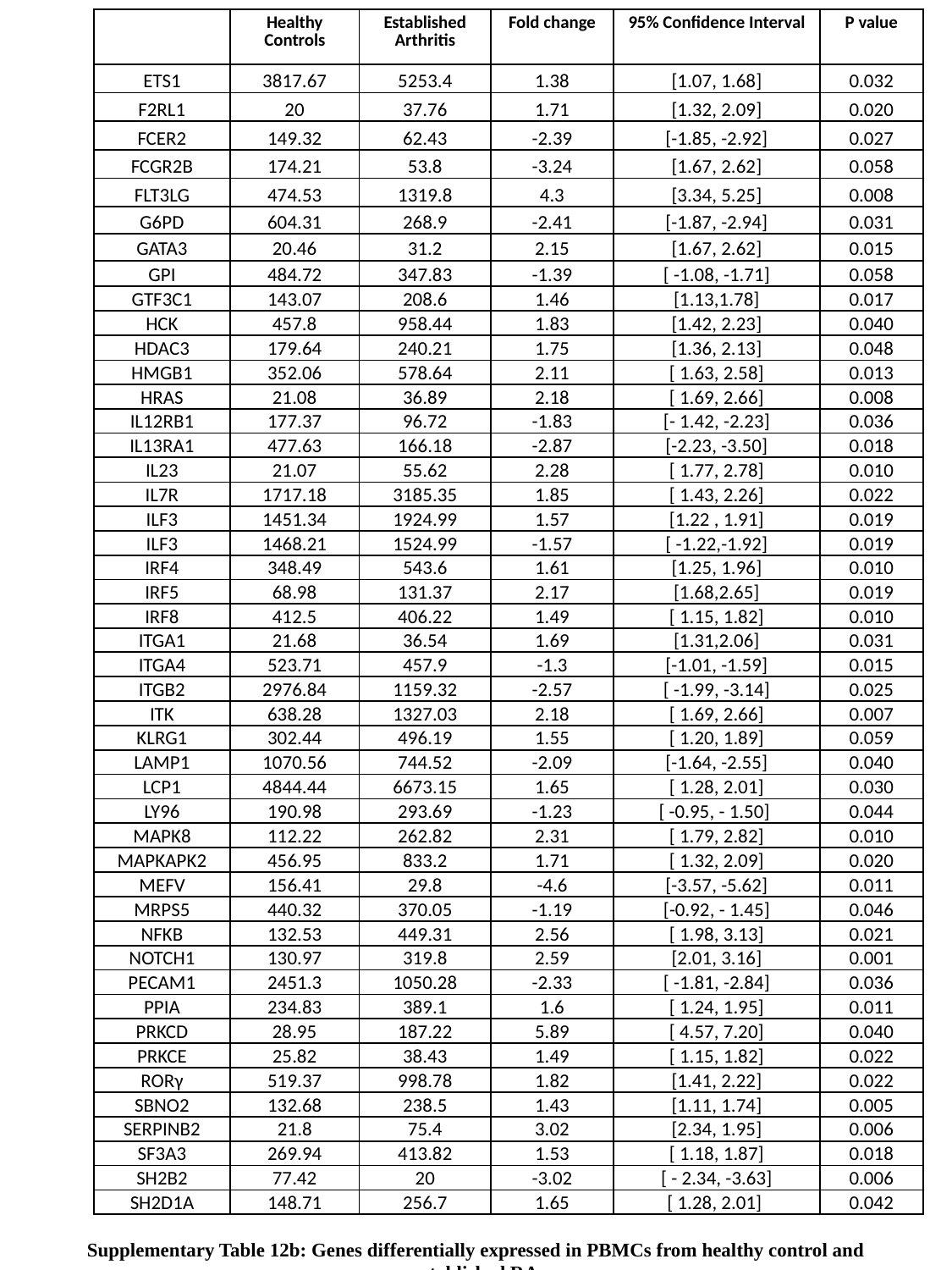

| | Healthy Controls | Established Arthritis | Fold change | 95% Confidence Interval | P value |
| --- | --- | --- | --- | --- | --- |
| ETS1 | 3817.67 | 5253.4 | 1.38 | [1.07, 1.68] | 0.032 |
| F2RL1 | 20 | 37.76 | 1.71 | [1.32, 2.09] | 0.020 |
| FCER2 | 149.32 | 62.43 | -2.39 | [-1.85, -2.92] | 0.027 |
| FCGR2B | 174.21 | 53.8 | -3.24 | [1.67, 2.62] | 0.058 |
| FLT3LG | 474.53 | 1319.8 | 4.3 | [3.34, 5.25] | 0.008 |
| G6PD | 604.31 | 268.9 | -2.41 | [-1.87, -2.94] | 0.031 |
| GATA3 | 20.46 | 31.2 | 2.15 | [1.67, 2.62] | 0.015 |
| GPI | 484.72 | 347.83 | -1.39 | [ -1.08, -1.71] | 0.058 |
| GTF3C1 | 143.07 | 208.6 | 1.46 | [1.13,1.78] | 0.017 |
| HCK | 457.8 | 958.44 | 1.83 | [1.42, 2.23] | 0.040 |
| HDAC3 | 179.64 | 240.21 | 1.75 | [1.36, 2.13] | 0.048 |
| HMGB1 | 352.06 | 578.64 | 2.11 | [ 1.63, 2.58] | 0.013 |
| HRAS | 21.08 | 36.89 | 2.18 | [ 1.69, 2.66] | 0.008 |
| IL12RB1 | 177.37 | 96.72 | -1.83 | [- 1.42, -2.23] | 0.036 |
| IL13RA1 | 477.63 | 166.18 | -2.87 | [-2.23, -3.50] | 0.018 |
| IL23 | 21.07 | 55.62 | 2.28 | [ 1.77, 2.78] | 0.010 |
| IL7R | 1717.18 | 3185.35 | 1.85 | [ 1.43, 2.26] | 0.022 |
| ILF3 | 1451.34 | 1924.99 | 1.57 | [1.22 , 1.91] | 0.019 |
| ILF3 | 1468.21 | 1524.99 | -1.57 | [ -1.22,-1.92] | 0.019 |
| IRF4 | 348.49 | 543.6 | 1.61 | [1.25, 1.96] | 0.010 |
| IRF5 | 68.98 | 131.37 | 2.17 | [1.68,2.65] | 0.019 |
| IRF8 | 412.5 | 406.22 | 1.49 | [ 1.15, 1.82] | 0.010 |
| ITGA1 | 21.68 | 36.54 | 1.69 | [1.31,2.06] | 0.031 |
| ITGA4 | 523.71 | 457.9 | -1.3 | [-1.01, -1.59] | 0.015 |
| ITGB2 | 2976.84 | 1159.32 | -2.57 | [ -1.99, -3.14] | 0.025 |
| ITK | 638.28 | 1327.03 | 2.18 | [ 1.69, 2.66] | 0.007 |
| KLRG1 | 302.44 | 496.19 | 1.55 | [ 1.20, 1.89] | 0.059 |
| LAMP1 | 1070.56 | 744.52 | -2.09 | [-1.64, -2.55] | 0.040 |
| LCP1 | 4844.44 | 6673.15 | 1.65 | [ 1.28, 2.01] | 0.030 |
| LY96 | 190.98 | 293.69 | -1.23 | [ -0.95, - 1.50] | 0.044 |
| MAPK8 | 112.22 | 262.82 | 2.31 | [ 1.79, 2.82] | 0.010 |
| MAPKAPK2 | 456.95 | 833.2 | 1.71 | [ 1.32, 2.09] | 0.020 |
| MEFV | 156.41 | 29.8 | -4.6 | [-3.57, -5.62] | 0.011 |
| MRPS5 | 440.32 | 370.05 | -1.19 | [-0.92, - 1.45] | 0.046 |
| NFKB | 132.53 | 449.31 | 2.56 | [ 1.98, 3.13] | 0.021 |
| NOTCH1 | 130.97 | 319.8 | 2.59 | [2.01, 3.16] | 0.001 |
| PECAM1 | 2451.3 | 1050.28 | -2.33 | [ -1.81, -2.84] | 0.036 |
| PPIA | 234.83 | 389.1 | 1.6 | [ 1.24, 1.95] | 0.011 |
| PRKCD | 28.95 | 187.22 | 5.89 | [ 4.57, 7.20] | 0.040 |
| PRKCE | 25.82 | 38.43 | 1.49 | [ 1.15, 1.82] | 0.022 |
| RORγ | 519.37 | 998.78 | 1.82 | [1.41, 2.22] | 0.022 |
| SBNO2 | 132.68 | 238.5 | 1.43 | [1.11, 1.74] | 0.005 |
| SERPINB2 | 21.8 | 75.4 | 3.02 | [2.34, 1.95] | 0.006 |
| SF3A3 | 269.94 | 413.82 | 1.53 | [ 1.18, 1.87] | 0.018 |
| SH2B2 | 77.42 | 20 | -3.02 | [ - 2.34, -3.63] | 0.006 |
| SH2D1A | 148.71 | 256.7 | 1.65 | [ 1.28, 2.01] | 0.042 |
Supplementary Table 12b: Genes differentially expressed in PBMCs from healthy control and established RA

## Slide 16
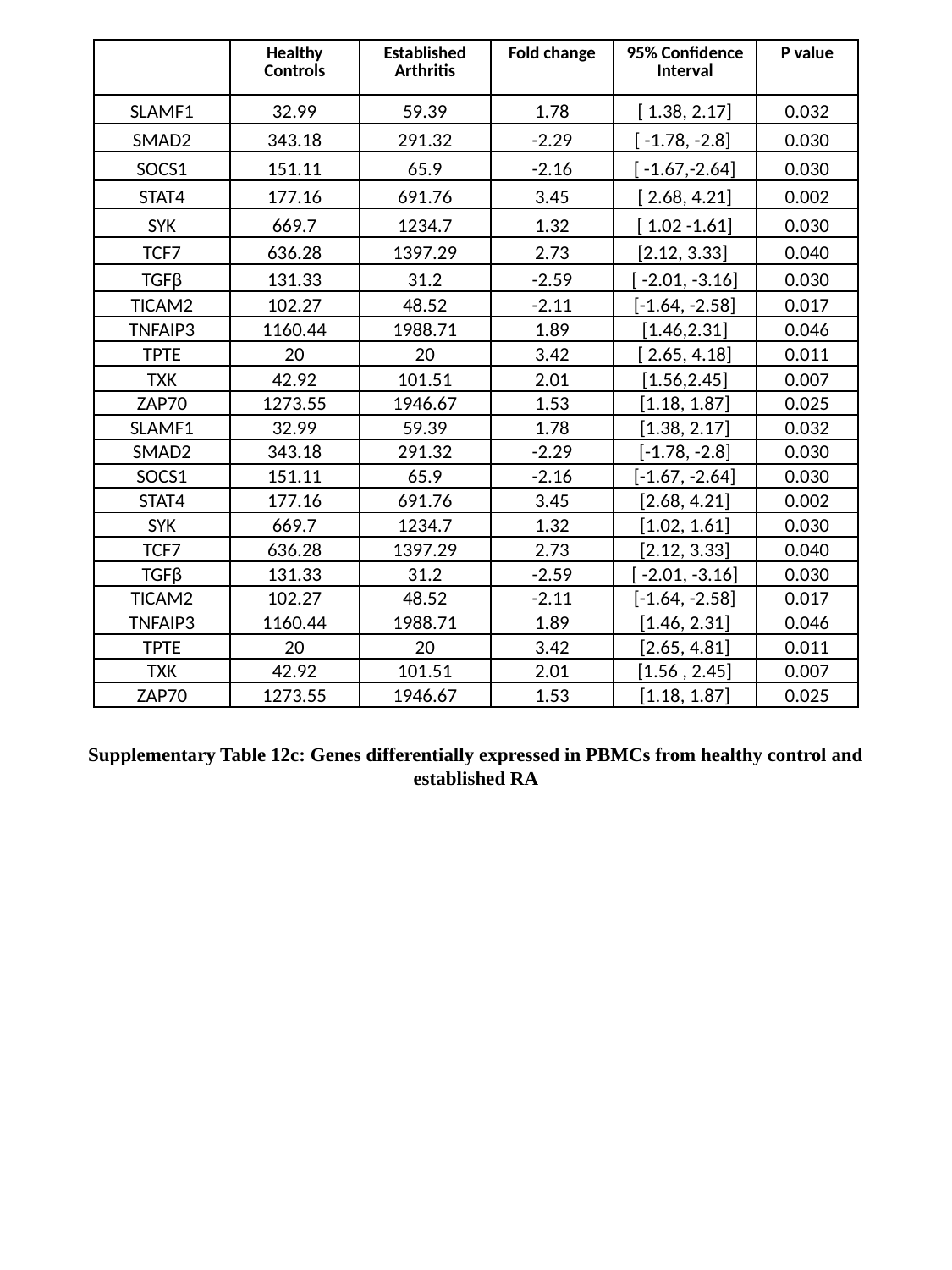

| | Healthy Controls | Established Arthritis | Fold change | 95% Confidence Interval | P value |
| --- | --- | --- | --- | --- | --- |
| SLAMF1 | 32.99 | 59.39 | 1.78 | [ 1.38, 2.17] | 0.032 |
| SMAD2 | 343.18 | 291.32 | -2.29 | [ -1.78, -2.8] | 0.030 |
| SOCS1 | 151.11 | 65.9 | -2.16 | [ -1.67,-2.64] | 0.030 |
| STAT4 | 177.16 | 691.76 | 3.45 | [ 2.68, 4.21] | 0.002 |
| SYK | 669.7 | 1234.7 | 1.32 | [ 1.02 -1.61] | 0.030 |
| TCF7 | 636.28 | 1397.29 | 2.73 | [2.12, 3.33] | 0.040 |
| TGFβ | 131.33 | 31.2 | -2.59 | [ -2.01, -3.16] | 0.030 |
| TICAM2 | 102.27 | 48.52 | -2.11 | [-1.64, -2.58] | 0.017 |
| TNFAIP3 | 1160.44 | 1988.71 | 1.89 | [1.46,2.31] | 0.046 |
| TPTE | 20 | 20 | 3.42 | [ 2.65, 4.18] | 0.011 |
| TXK | 42.92 | 101.51 | 2.01 | [1.56,2.45] | 0.007 |
| ZAP70 | 1273.55 | 1946.67 | 1.53 | [1.18, 1.87] | 0.025 |
| SLAMF1 | 32.99 | 59.39 | 1.78 | [1.38, 2.17] | 0.032 |
| SMAD2 | 343.18 | 291.32 | -2.29 | [-1.78, -2.8] | 0.030 |
| SOCS1 | 151.11 | 65.9 | -2.16 | [-1.67, -2.64] | 0.030 |
| STAT4 | 177.16 | 691.76 | 3.45 | [2.68, 4.21] | 0.002 |
| SYK | 669.7 | 1234.7 | 1.32 | [1.02, 1.61] | 0.030 |
| TCF7 | 636.28 | 1397.29 | 2.73 | [2.12, 3.33] | 0.040 |
| TGFβ | 131.33 | 31.2 | -2.59 | [ -2.01, -3.16] | 0.030 |
| TICAM2 | 102.27 | 48.52 | -2.11 | [-1.64, -2.58] | 0.017 |
| TNFAIP3 | 1160.44 | 1988.71 | 1.89 | [1.46, 2.31] | 0.046 |
| TPTE | 20 | 20 | 3.42 | [2.65, 4.81] | 0.011 |
| TXK | 42.92 | 101.51 | 2.01 | [1.56 , 2.45] | 0.007 |
| ZAP70 | 1273.55 | 1946.67 | 1.53 | [1.18, 1.87] | 0.025 |
Supplementary Table 12c: Genes differentially expressed in PBMCs from healthy control and established RA

## Slide 17
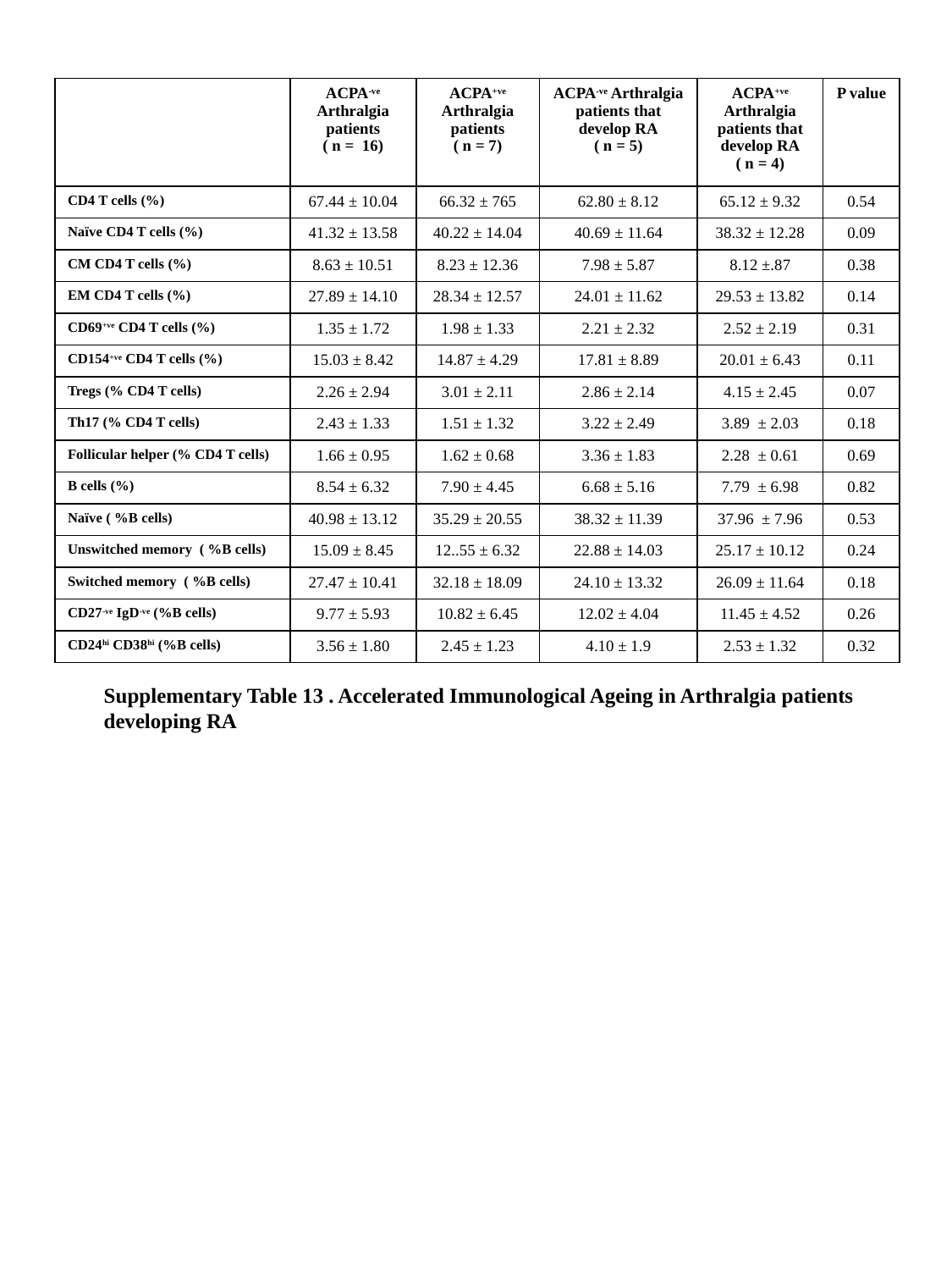

| | ACPA-ve Arthralgia patients ( n = 16) | ACPA+ve Arthralgia patients ( n = 7) | ACPA-ve Arthralgia patients that develop RA ( n = 5) | ACPA+ve Arthralgia patients that develop RA ( n = 4) | P value |
| --- | --- | --- | --- | --- | --- |
| CD4 T cells (%) | 67.44 ± 10.04 | 66.32 ± 765 | 62.80 ± 8.12 | 65.12 ± 9.32 | 0.54 |
| Naïve CD4 T cells (%) | 41.32 ± 13.58 | 40.22 ± 14.04 | 40.69 ± 11.64 | 38.32 ± 12.28 | 0.09 |
| CM CD4 T cells (%) | 8.63 ± 10.51 | 8.23 ± 12.36 | 7.98 ± 5.87 | 8.12 ±.87 | 0.38 |
| EM CD4 T cells (%) | 27.89 ± 14.10 | 28.34 ± 12.57 | 24.01 ± 11.62 | 29.53 ± 13.82 | 0.14 |
| CD69+ve CD4 T cells (%) | 1.35 ± 1.72 | 1.98 ± 1.33 | 2.21 ± 2.32 | 2.52 ± 2.19 | 0.31 |
| CD154+ve CD4 T cells (%) | 15.03 ± 8.42 | 14.87 ± 4.29 | 17.81 ± 8.89 | 20.01 ± 6.43 | 0.11 |
| Tregs (% CD4 T cells) | 2.26 ± 2.94 | 3.01 ± 2.11 | 2.86 ± 2.14 | 4.15 ± 2.45 | 0.07 |
| Th17 (% CD4 T cells) | 2.43 ± 1.33 | 1.51 ± 1.32 | 3.22 ± 2.49 | 3.89 ± 2.03 | 0.18 |
| Follicular helper (% CD4 T cells) | 1.66 ± 0.95 | 1.62 ± 0.68 | 3.36 ± 1.83 | 2.28 ± 0.61 | 0.69 |
| B cells (%) | 8.54 ± 6.32 | 7.90 ± 4.45 | 6.68 ± 5.16 | 7.79 ± 6.98 | 0.82 |
| Naïve ( %B cells) | 40.98 ± 13.12 | 35.29 ± 20.55 | 38.32 ± 11.39 | 37.96 ± 7.96 | 0.53 |
| Unswitched memory ( %B cells) | 15.09 ± 8.45 | 12..55 ± 6.32 | 22.88 ± 14.03 | 25.17 ± 10.12 | 0.24 |
| Switched memory ( %B cells) | 27.47 ± 10.41 | 32.18 ± 18.09 | 24.10 ± 13.32 | 26.09 ± 11.64 | 0.18 |
| CD27-ve IgD-ve (%B cells) | 9.77 ± 5.93 | 10.82 ± 6.45 | 12.02 ± 4.04 | 11.45 ± 4.52 | 0.26 |
| CD24hi CD38hi (%B cells) | 3.56 ± 1.80 | 2.45 ± 1.23 | 4.10 ± 1.9 | 2.53 ± 1.32 | 0.32 |
Supplementary Table 13 . Accelerated Immunological Ageing in Arthralgia patients developing RA

## Slide 18
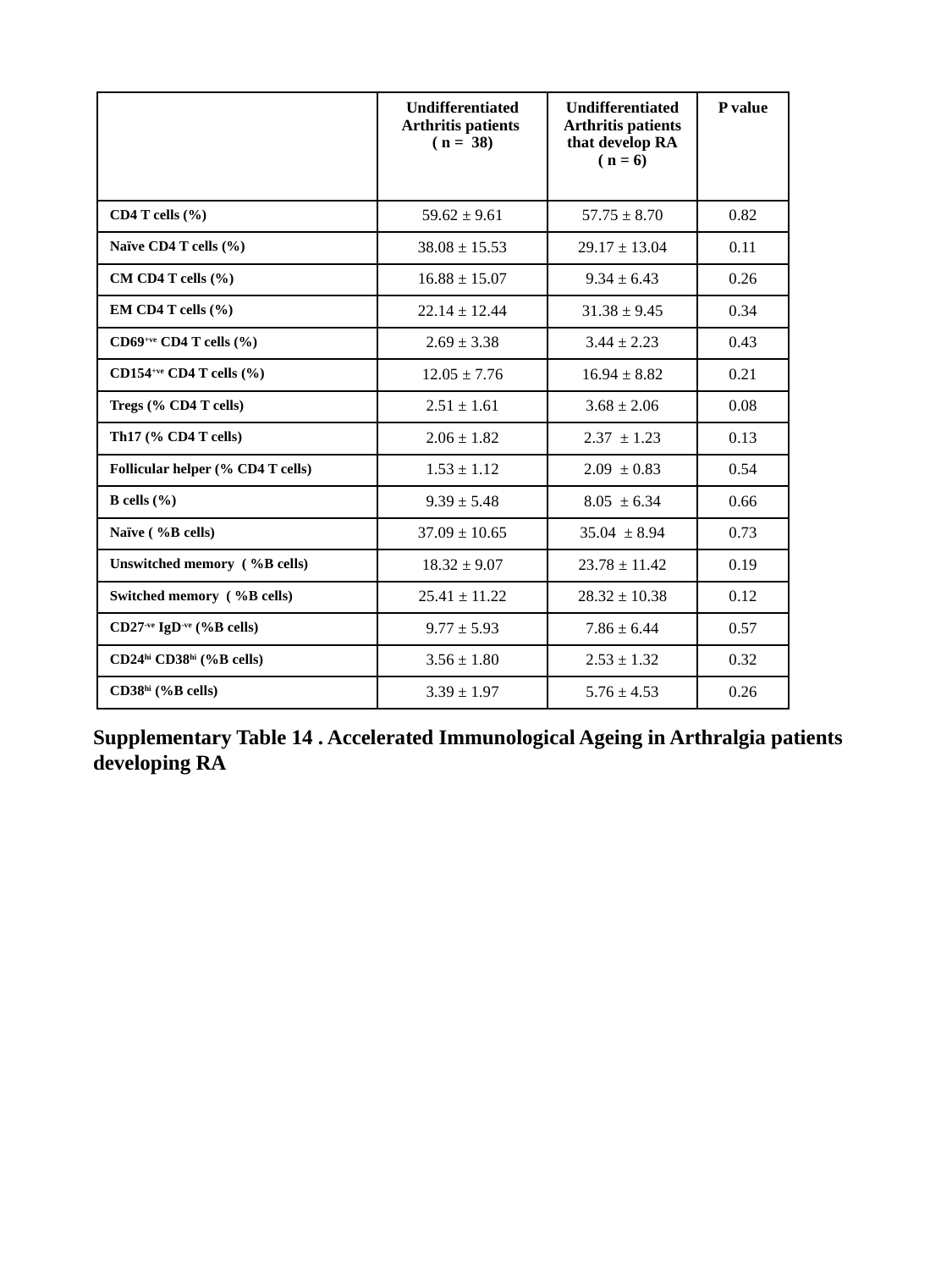

| | Undifferentiated Arthritis patients ( n = 38) | Undifferentiated Arthritis patients that develop RA ( n = 6) | P value |
| --- | --- | --- | --- |
| CD4 T cells (%) | 59.62 ± 9.61 | 57.75 ± 8.70 | 0.82 |
| Naïve CD4 T cells (%) | 38.08 ± 15.53 | 29.17 ± 13.04 | 0.11 |
| CM CD4 T cells (%) | 16.88 ± 15.07 | 9.34 ± 6.43 | 0.26 |
| EM CD4 T cells (%) | 22.14 ± 12.44 | 31.38 ± 9.45 | 0.34 |
| CD69+ve CD4 T cells (%) | 2.69 ± 3.38 | 3.44 ± 2.23 | 0.43 |
| CD154+ve CD4 T cells (%) | 12.05 ± 7.76 | 16.94 ± 8.82 | 0.21 |
| Tregs (% CD4 T cells) | 2.51 ± 1.61 | 3.68 ± 2.06 | 0.08 |
| Th17 (% CD4 T cells) | 2.06 ± 1.82 | 2.37 ± 1.23 | 0.13 |
| Follicular helper (% CD4 T cells) | 1.53 ± 1.12 | 2.09 ± 0.83 | 0.54 |
| B cells (%) | 9.39 ± 5.48 | 8.05 ± 6.34 | 0.66 |
| Naïve ( %B cells) | 37.09 ± 10.65 | 35.04 ± 8.94 | 0.73 |
| Unswitched memory ( %B cells) | 18.32 ± 9.07 | 23.78 ± 11.42 | 0.19 |
| Switched memory ( %B cells) | 25.41 ± 11.22 | 28.32 ± 10.38 | 0.12 |
| CD27-ve IgD-ve (%B cells) | 9.77 ± 5.93 | 7.86 ± 6.44 | 0.57 |
| CD24hi CD38hi (%B cells) | 3.56 ± 1.80 | 2.53 ± 1.32 | 0.32 |
| CD38hi (%B cells) | 3.39 ± 1.97 | 5.76 ± 4.53 | 0.26 |
Supplementary Table 14 . Accelerated Immunological Ageing in Arthralgia patients developing RA

## Slide 19
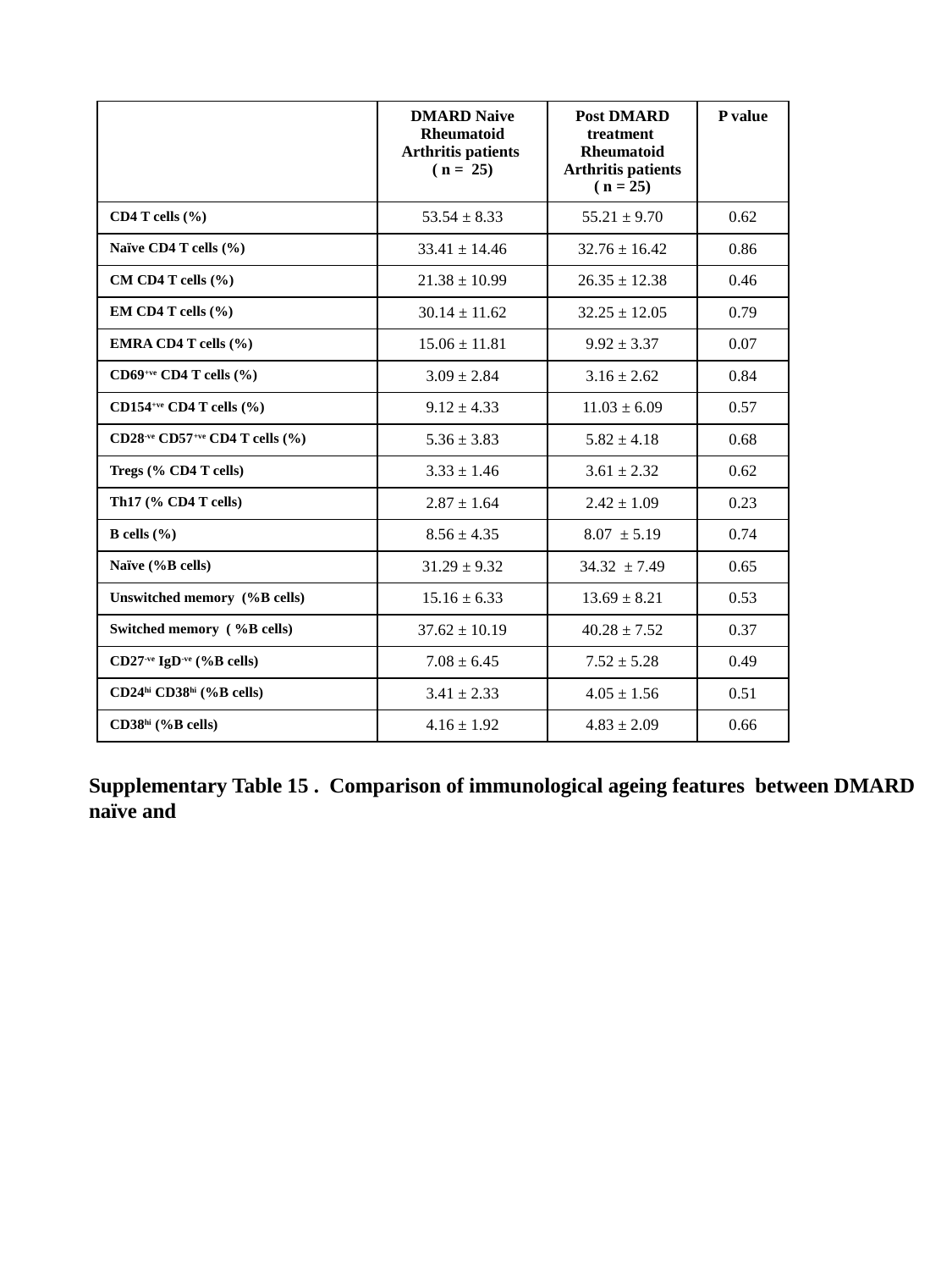

| | DMARD Naive Rheumatoid Arthritis patients ( n = 25) | Post DMARD treatment Rheumatoid Arthritis patients ( n = 25) | P value |
| --- | --- | --- | --- |
| CD4 T cells (%) | 53.54 ± 8.33 | 55.21 ± 9.70 | 0.62 |
| Naïve CD4 T cells (%) | 33.41 ± 14.46 | 32.76 ± 16.42 | 0.86 |
| CM CD4 T cells (%) | 21.38 ± 10.99 | 26.35 ± 12.38 | 0.46 |
| EM CD4 T cells (%) | 30.14 ± 11.62 | 32.25 ± 12.05 | 0.79 |
| EMRA CD4 T cells (%) | 15.06 ± 11.81 | 9.92 ± 3.37 | 0.07 |
| CD69+ve CD4 T cells (%) | 3.09 ± 2.84 | 3.16 ± 2.62 | 0.84 |
| CD154+ve CD4 T cells (%) | 9.12 ± 4.33 | 11.03 ± 6.09 | 0.57 |
| CD28-ve CD57+ve CD4 T cells (%) | 5.36 ± 3.83 | 5.82 ± 4.18 | 0.68 |
| Tregs (% CD4 T cells) | 3.33 ± 1.46 | 3.61 ± 2.32 | 0.62 |
| Th17 (% CD4 T cells) | 2.87 ± 1.64 | 2.42 ± 1.09 | 0.23 |
| B cells (%) | 8.56 ± 4.35 | 8.07 ± 5.19 | 0.74 |
| Naïve (%B cells) | 31.29 ± 9.32 | 34.32 ± 7.49 | 0.65 |
| Unswitched memory (%B cells) | 15.16 ± 6.33 | 13.69 ± 8.21 | 0.53 |
| Switched memory ( %B cells) | 37.62 ± 10.19 | 40.28 ± 7.52 | 0.37 |
| CD27-ve IgD-ve (%B cells) | 7.08 ± 6.45 | 7.52 ± 5.28 | 0.49 |
| CD24hi CD38hi (%B cells) | 3.41 ± 2.33 | 4.05 ± 1.56 | 0.51 |
| CD38hi (%B cells) | 4.16 ± 1.92 | 4.83 ± 2.09 | 0.66 |
Supplementary Table 15 . Comparison of immunological ageing features between DMARD naïve and
